# Mesenchymal stem cells-derived extracellular vesicles protect against oxidative stress-induced xenogeneic biological root injury via adaptive regulation of the PI3K/Akt/NRF2 pathway

**DOI:** 10.1186/s12951-023-02214-5

**Published:** 2023-12-04

**Authors:** Haojie Fu, Lin Sen, Fangqi Zhang, Sirui Liu, Meiyue Wang, Hongyan Mi, Mengzhe Liu, Bingyan Li, Shumin Peng, Zelong Hu, Jingjing Sun, Rui Li

**Affiliations:** 1 Department of Stomatology, The First Affiliated Hospital of Zhengzhou University, Zhengzhou, 45000 China; 2 Academy of Medical Sciences at Zhengzhou University, Zhengzhou, 45000 China

**Keywords:** Biological tooth root, Extracellular vesicle, Oxidative stress, Extracellular matrix, Dental follicle cell

## Abstract

Xenogeneic extracellular matrices (xECM) for cell support have emerged as a potential strategy for addressing the scarcity of donor matrices for allotransplantation. However, the poor survival rate or failure of xECM-based organ transplantation is due to the negative impacts of high-level oxidative stress and inflammation on seed cell viability and stemness. Herein, we constructed xenogeneic bioengineered tooth roots (bio-roots) and used extracellular vesicles from human adipose-derived mesenchymal stem cells (hASC-EVs) to shield bio-roots from oxidative damage. Pretreatment with hASC-EVs reduced cell apoptosis, reactive oxygen species generation, mitochondrial changes, and DNA damage. Furthermore, hASC-EV treatment improved cell proliferation, antioxidant capacity, and odontogenic and osteogenic differentiation, while significantly suppressing oxidative damage by activating the phosphatidylinositol 3-kinase (PI3K)/Akt pathway and nuclear factor erythroid 2 (NFE2)-related factor 2 (NRF2) nuclear translocation via p62-associated Kelch-like ECH-associated protein 1 (KEAP1) degradation. Inhibition of PI3K/Akt and *Nrf2* knockdown reduced antioxidant capacity, indicating that the PI3K/Akt/NRF2 pathway partly mediates these effects. In subcutaneous grafting experiments using Sprague–Dawley rats, hASC-EV administration significantly enhanced the antioxidant effect of the bio-root, improved the regeneration efficiency of periodontal ligament-like tissue, and maximized xenograft function. Conclusively, therefore, hASC-EVs have the potential to be used as an immune modulator and antioxidant for treating oxidative stress-induced bio-root resorption and degradation, which may be utilized for the generation and restoration of other intricate tissues and organs.

**Graphic Abstract:**

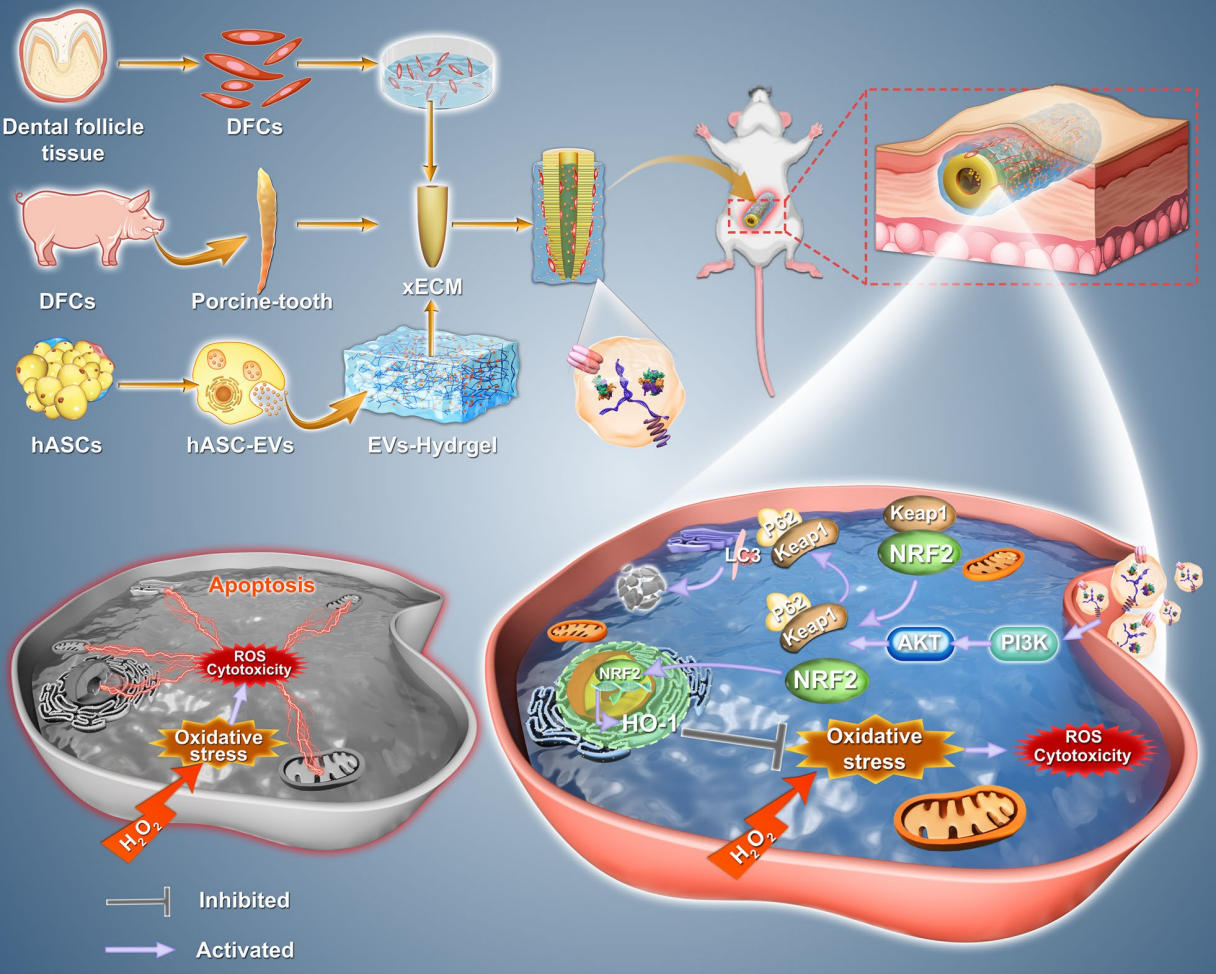

**Supplementary Information:**

The online version contains supplementary material available at 10.1186/s12951-023-02214-5.

## Introduction

The extracellular matrix (ECM) is a tissue-specific 3D network of fibrillar proteins, proteoglycans, and glycosaminoglycans that provides cells and tissues with spatial communication signals, structural and ductile properties, and a medium for the transport and movement of nutrients and oxygen [1–3]. Owing to the limited donor pool of human-derived ECM, xenogeneic ECM (xECM) is currently being studied for application in tissue and organ regeneration [5, 6]. Porcine tooth-derived dentin matrix, a type of xECM with a histological structure and bioactive factors comparable to those of human-derived dentin matrix, can promote odontogenesis and biomineralization [7]. xECM composite odontogenic stem cells have been used to construct bio-roots while regenerating dentin structure, dental pulp-like tissue, and periodontal ligament (PDL)-like tissue [8, 9]. However, immunological rejection of xenogeneic-treated dentin matrix results in a high level oxidative stress culminates in the disruption of intercellular and cell–ECM adhesions, ultimately leading to graft failure [10]. Consequently, this process culminates in the disruption of intercellular and cell–ECM adhesions, ultimately leading to graft failure [11–13].

Oxidative stress usually occurs due to the production of oxidants in cells to a level that exceeds endogenous antioxidant capacity. Several studies have identified significant connections between oxidative stress, inflammation and healing, the major events connected to the fate of implanted biomaterials. Although oxidative stress is essential for normal cell function [14], excessive accumulation of oxidants (such as ROS, reactive nitrogen species, and lipid peroxidation) can cause considerable oxidative damage, including DNA damage, oxidation of cellular proteins, lipid peroxidation damage, and mitochondrial dysfunction, which are detrimental to cell survival and tissue regeneration, results in graft dysfunction and failure [15–17]. Hence, the successful integration of biomaterials necessitates a careful assessment of the equilibrium between oxidants and antioxidants. These findings suggest that augmenting the antioxidant capacity of the graft could serve as a viable approach to alleviate the detrimental consequences of oxidative stress after material transplantation [18–20].

Extracellular vesicles (EVs), a major output of the paracrine pathway, are bilipid-layered, nano-dimensional microvesicles that encapsulate various bioactive substances, including proteins, DNA, and microRNAs (miRNAs), and have similar biological effects as their cells of origin [21–23]. hASC-EVs are envisioned as a promising and competent tool for the forthcoming era of therapeutic platforms for regenerative medicine because of their capacity to successfully alter the physiological and pathological activities of recipient cells by delivering their biologically active cargo [24–27]. In addition to directly promoting tissue and organ regeneration, EVs can be involved in regulating microenvironments that are detrimental to tissue damage repair [28]. Several studies have found that EVs can reduce ischemia–reperfusion injury by inhibiting oxidative stress-induced endothelial cell senescence and inhibiting oxidative damage in osteoarthritis [28–30]. Investigating ways to improve the microenvironment during oxidative stress caused by immune rejection after allogeneic material transplantation through the use of EVs is therefore an enticing avenue of research, although the impact of EVs on xECM-based xenotransplantation remains unknown.

Nuclear factor erythroid 2 (NFE2)-related factor 2 (NRF2) functions as an endogenous regulator of the cellular antioxidant defense system, governing various genes associated with antioxidant and cytoprotective activities [31]. During instances of oxidative stress, NRF2 is liberated from the NRF2-Kelch-like ECH -associated protein 1 (KEAP1) complex via p62/sequestosome 1 (SQSTM1), also known as P62, which competitively binds to KEAP1. Subsequently, the NRF2 protein undergoes translocation to the nucleus, where it initiates the transcriptional activation of the downstream target gene heme oxygenase-1 (*HO-1*) [32]. The phosphatidylinositol 3-kinase (PI3K)/Akt pathway, which regulates several biological processes such as cellular biosynthesis, energy metabolism, and differentiation, is an essential upstream regulator of NRF2 [33–36]. EVs can provide neuroprotection, promote angiogenesis, improve cartilage and bone regeneration, and transplant cellular senescence by activating the PI3K/Akt pathway [37–39]. Therefore, hASC-EVs may protect bio-roots against oxidative stress by activating the PI3K/ATK/NRF2 pathway.

Here, we examined the efficacy of hASC-EVs in ameliorating oxidative stress-induced damage in dental follicle cells (DFCs) in the xenogeneic bio-root system. We stimulated cells with hydrogen peroxide (H_2_O_2_) to simulate the in vivo oxidative stress environment of DFCs on the surface of xenogeneic bio-roots and explored the potential molecular mechanisms of treatment with the PI3K/Akt inhibitor LY294002 and *Nrf2* knockdown in the DFCs. The animal model was mainly used to detect xenogeneic bio-root growth by subcutaneous grafting in Sprague–Dawley (SD) rats. Furthermore, because the direct injection of EV solution led to rapid EV depletion and a low retention rate, we utilized hydrogel-loaded hASC-EVs to encapsulate the bio-roots to extend the efficacy of hASC-EVs in xenogeneic bio-roots.

## Results

### Characterization of DFCs

Primary cells were observed after dental follicle tissue culture for 3 d (Additional file 1: Fig. S1a). The divided cells had typical polygonal and spindle shapes along with the ability to form colonies. ARS and Oil Red O staining revealed mineralized nodules and lipid droplets, respectively, indicating that DFCs are capable of osteogenic and adipogenic differentiation. The mesenchymal stem cell (MSC) markers c-Kit, stro-1, and vimentin were present, as indicated by the positive results of the DFCs, whereas the CK-14 epithelial cell marker was absent (Additional file 1: Fig. S1b). Flow cytometric analysis indicated that DFCs expressed the markers CD105, CD90, and CD73 but lacked expression of the hematopoietic lineage antigens CD31 and CD34 (Additional file 1: Fig. S1c).

### Characterization of hASC-derived EVs

**Fig. 1:**
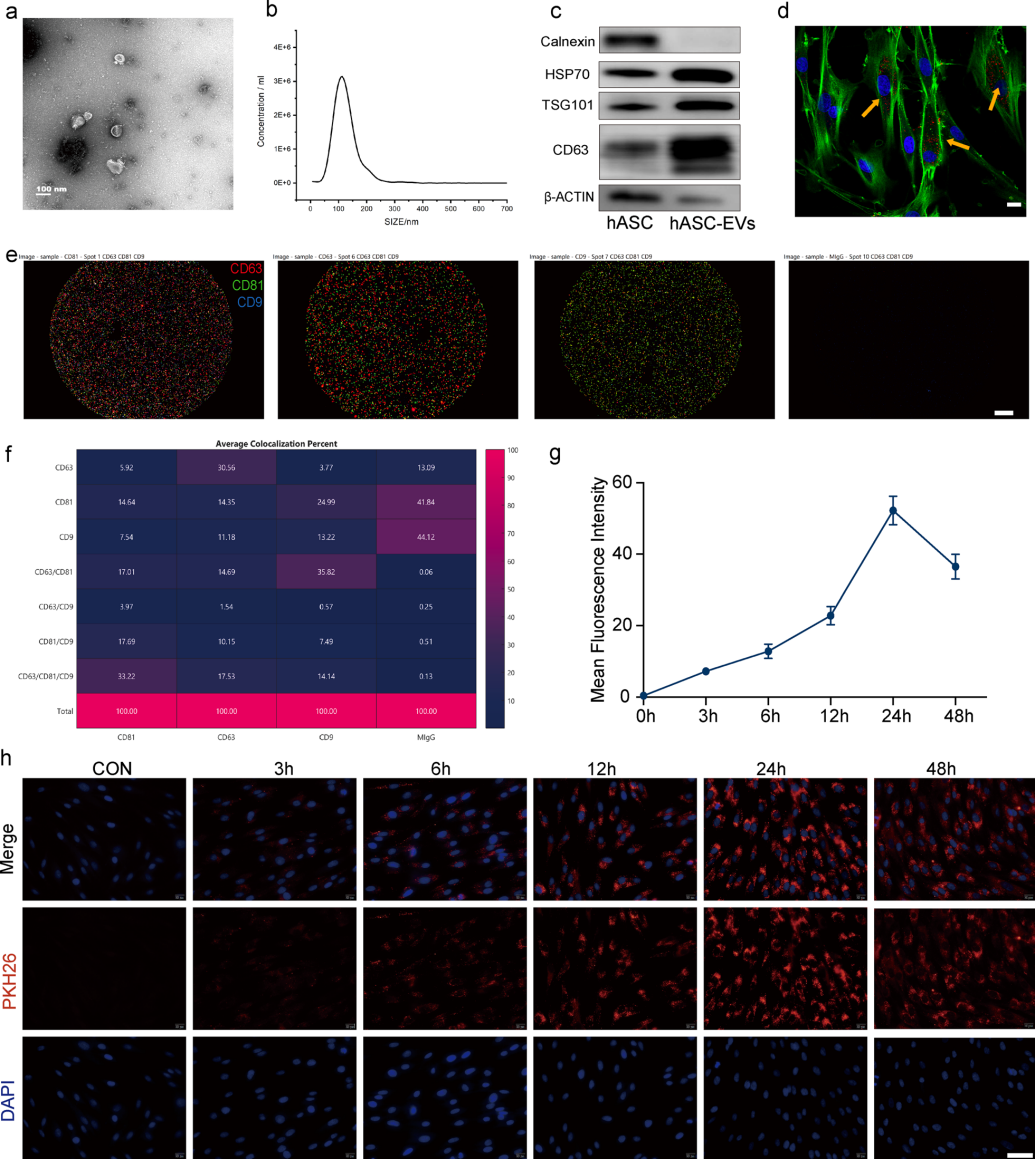
Characterization of extracellular vesicles from human adipose-derived mesenchymal stem cell extracellular vesicles (hASC-EVs). **a** Transmission electron microscopy (TEM) shows the classic “rim of a cup” and particle vesicle shape. **b** Nanoparticle tracking analysis (NTA) shows the EV diameter. **c** Western blot analysis of EVs shows positive results for CD63, TSG101, and HSP70 and negative results for Calnexin. **d** hASC-EVs taken in by dental follicle cells. The yellow arrow points to PKH26-labeled hASC-EVs. Red: PKH26-labeled EVs; green: FITC-phalloidin; blue: DAPI. Scale bars = 20 µm. **e**, **f** ExoView showing CD9, CD63, and CD81 expression in hASC-EVs. Scale bars = 20 μm. **g** The line graph shows the mean fluorescence intensity (MFI) change of PKH26. (h) PKH26 labeling of EVs shows red coloration, observed microscopically at 0, 3, 6, 12, 24, and 48 h. Scale bars = 50 µm

The transmission electron microscopy assessment revealed that the vesicles exhibited a characteristic morphology resembling the shape of a cup's rim, with the presence of granules (Fig. 1a). Nanoparticle tracking analysis was employed to investigate the size distribution of hASC-EVs. The majority of these EVs exhibited the anticipated diameter of 117 nm, as depicted in Fig. 1b. The results of the western blot analysis demonstrated that the hASC-EVs exhibited positive expression of CD63, TSG101, and HSP70, whereas Calnexin was not detected (Fig. 1c). The hASC-EVs labeled with PKH26 were internalized by DFCs within a time frame of 3 h after being introduced into the culture medium (Fig. 1d). Furthermore, the ExoView technique provided evidence of the presence of the well-established EV markers CD9, CD63, and CD81 within the EVs, as depicted in Fig. 1e and f [40, 41].

### hASC-EV labeling and uptake by DFCs

To confirm whether hASC-EVs can be efficiently endocytosed by DFCs, we incubated PKH26-labeled hASC-EVs with DFC at different time periods. Our data showed that PKH26-labeled hASC-EVs were taken up within 3 h after addition to the culture medium. Red fluorescence signals were detected in the DFCs over time (Fig. 1g). By 24 h, almost all DFCs exhibited red fluorescence, and the fluorescence intensity remained unchanged after 48 h (Fig. 1h). Thus, EVs can enter cells and potentially be used therapeutically to modulate function.

### Establishment of an H_2_O_2_-induced oxidative stress model

**Fig. 2:**
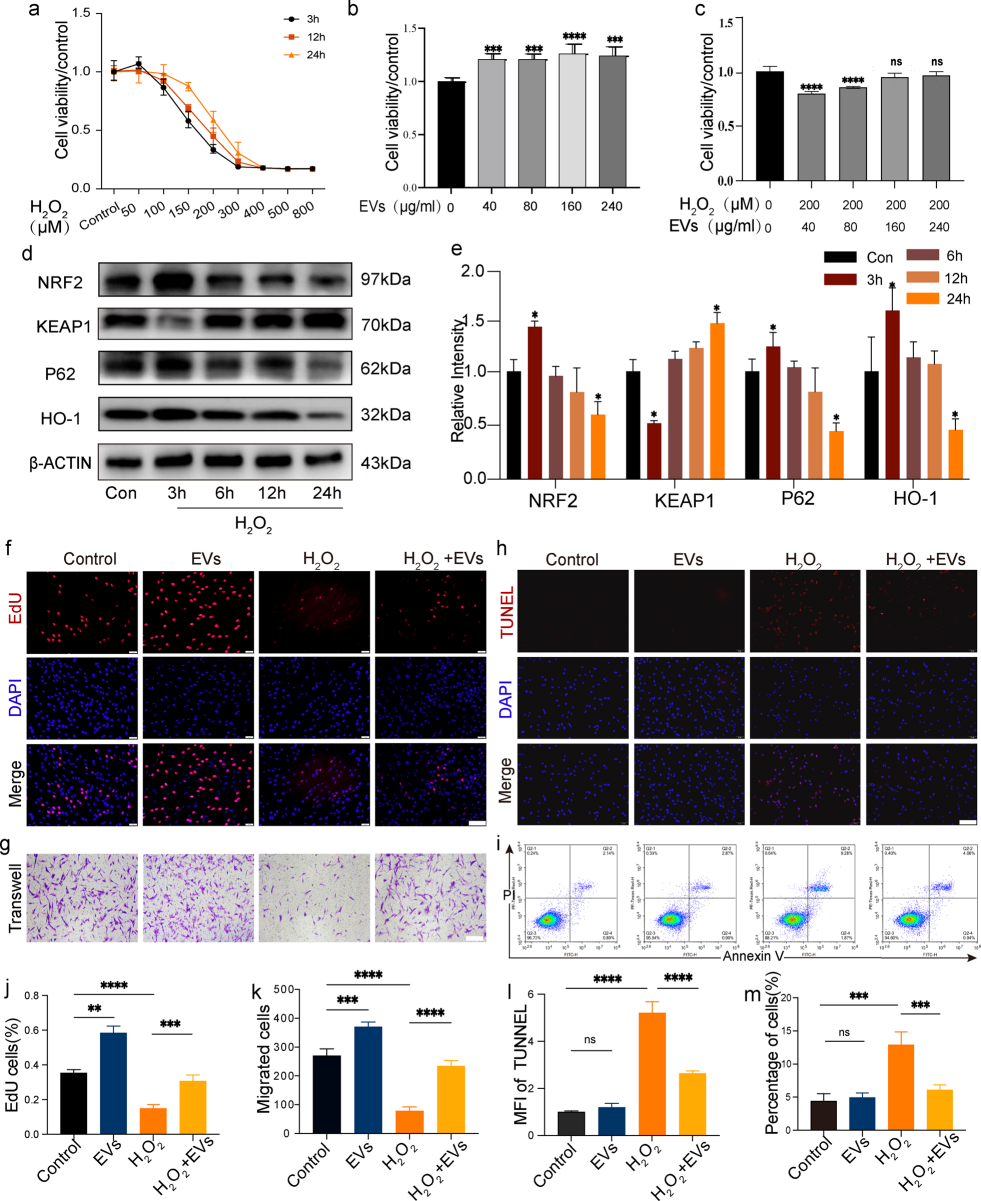
Effects of human adipose-derived mesenchymal stem cell extracellular vesicles (hASC-EVs) and H_2_O_2_ on dental follicle cell (DFC) activity, migration, and apoptosis. **a** Cell survival after the stimulation of DFCs with different H_2_O_2_ concentrations, measured using the CCK-8 assay. **b** Effects of various EV concentrations on cell proliferation were examined using the CCK-8 assay. **c** Effects of pretreatment with various EV concentrations on cell viability under H_2_O_2_ stimulation detected using the CCK-8 assay. **d** Western blot results showing changes in the NRF2 signaling pathway in DFCs at different periods following H_2_O_2_ stimulation. **e** Histogram illustrating the quantitative analysis of protein expression differences along the NRF2 signaling pathway between the groups. **f** Ethynyldeoxyuridine (EdU) staining fluorescence showing the effects of various treatments on cell proliferation. Scale bar = 100 μm. **g** Transwell assay-based detection of changes in cell migration ability. Scale bar = 100 μm. **h** TUNEL staining (red) to analyze DFC apoptosis; DAPI-stained cell nuclei (blue). Scale bar = 100 μm. **i** Annexin V/PI double-staining flow cytometry measured apoptosis in H_2_O_2_-stimulated DFCs following hASC-EV pretreatment. Histograms of EdU cells (**j**), migrated cells (**k**), mean fluorescence intensity (MFI) of TUNEL (**l**), and apoptosis percentage in cells (m) in each group. Not significant (NS) is indicated by *P* > 0.05, *P < 0.05, **P < 0.01, ***P < 0.001, and ****P < 0.0001, compared to the control

To examine cell survival after oxidative damage in vitro, DFC cultures were treated with 0, 50, 100, 200, 300, 400, 500, or 800 μM H_2_O_2_ for 3, 6, 12, and 24 h to model an oxidative stress environment. The survival rates of DFCs exhibited an ascending and then descending trend (Fig. 2a), with a considerable decrease in survival following treatment with H_2_O_2_ concentrations exceeding 200 μM. Considering that cell death may interfere with experimental data, a sublethal dosage of 200 μM H_2_O_2_ was used to develop the oxidative stress model in subsequent studies. Next, we explored the involvement of the NRF2/HO-1 signaling cascade in H_2_O_2_-induced oxidative stress. NRF2 was assessed via western blotting after the DFCs were treated with H_2_O_2_ for 3, 6, 12, and 24 h. The expression of NRF2, P62, and HO-1 initially increased at 12 h and then gradually declined at 24 h (Fig. 2d). However, KEAP1 levels decreased considerably at 3 h and continued to increase afterward (Fig. 2e), indicating that NRF2 protein expression may be controlled at two different periods.

### hASC-EVs alleviate oxidative stress-induced cytotoxicity and cell migration in DFCs

The proliferation of DFCs was assessed by conducting a CCK-8 assay to investigate the impact of different concentrations of hASC-EVs. As shown in Fig. 2b, hASC-EV concentrations > 160 μg/mL did not cause a further increase in cell proliferation. To further investigate the effect of hASC-EVs on H_2_O_2_-damaged cells, DFCs were incubated for 24 h with 200 μM H_2_O_2_ after being pretreated with various hASC-EV doses. The findings revealed that a concentration of 160 μg/mL was the optimal concentration for further investigations on the effects of hASC-EVs on H_2_O_2_-induced cell injury (Fig. 2c). Ethynyldeoxyuridine (EdU) experiments further demonstrated that hASC-EVs promoted cell proliferation and prevented oxidative damage to cellular functions (Fig. 2f and h). Furthermore, the administration of hASC-EVs resulted in enhanced migration of DFCs. The results obtained from the Transwell assay demonstrate a significant reduction in the quantity of migratory cells observed in the H_2_O_2_ experimental group compared to that in the control group. Conversely, the EV group exhibited a notable increase in cell migration relative to the group serving as the control (Fig. 2g and k). Pretreatment with hASC-EVs substantially reduced the cell migration inhibition caused by H_2_O_2_ in the EVs + H_2_O_2_ group, which indicated that hASC-EVs preserved the ability of DFCs to migrate under oxidative stress.

### hASC-EVs reduce H_2_O_2_-induced apoptosis in DFCs

The TUNEL assay was employed to evaluate the apoptosis of DFCs. The groups treated with H_2_O_2_ exhibited a greater quantity of TUNEL-positive cells compared to the control groups. Nevertheless, the number of TUNEL-positive cells induced by H_2_O_2_ decreased following pretreatment with hASC-EVs (Fig. 2h). Additionally, the flow cytometry findings exhibited a consistent pattern with the TUNEL staining results (Fig. 2i), suggesting that hASC-EVs mitigated cellular apoptosis in the presence of oxidative stress (Fig. 2l and m).

### hASC-EVs inhibit oxidative injury and alleviate oxidative responsiveness in H_2_O_2_-stimulated DFCs

**Fig. 3:**
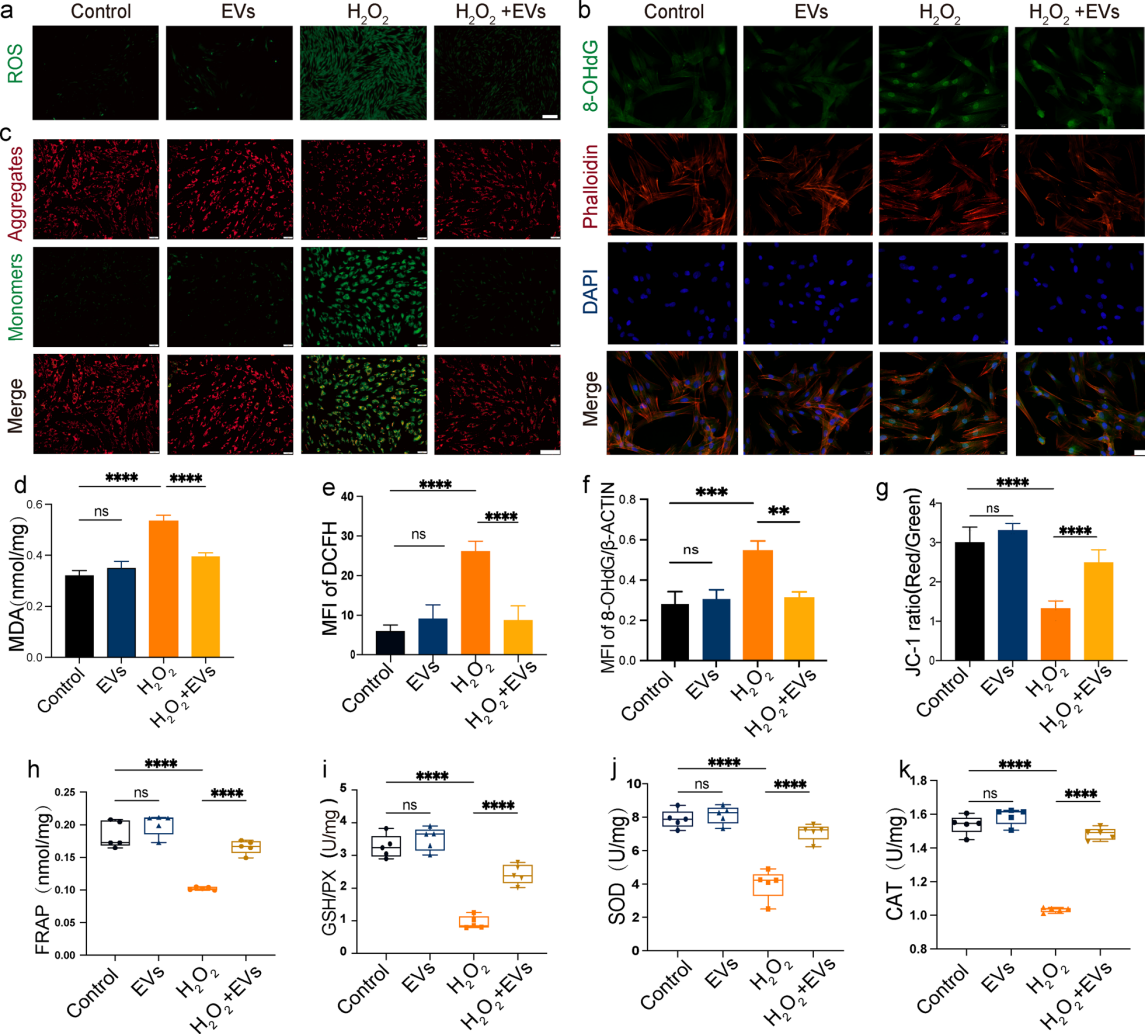
Human adipose-derived mesenchymal stem cell extracellular vesicles (hASC-EVs) inhibited oxidative damage, promoted antioxidant activity, and alleviated H_2_O_2_-stimulated oxidative reactivity in dental follicle cells (DFCs). The oxidative stress and antioxidant activity of the experimental group in response to H_2_O_2_ stimulation were examined after hASC-EV administration. **a** Representative images showing DCF (green) and reactive oxygen species (ROS) production in each group. Scale bar = 200 μm. **b** Representative images showing 8-OHdG (green nuclei) immunostaining results and the cytoskeleton (red) in each group. Scale bar = 50 μm. **c** Representative images showing the MMP of DFCs, detected using JC-1 staining, which was identified by green fluorescence for the monomeric form of JC-1 and red fluorescence for potential-dependent aggregation. Scale bar = 100 μm. **d** Histograms showing lipid peroxidation (MDA) concentrations in the different groups. **e**–**g** Histograms showing quantification of the mean fluorescence intensity (MFI) of DCFH (**e**), 8-OHdG (**f**), and JC-1 ratio (**g**) in DFCs. **h**–**k** Histograms showing the FRAP (**h**), GSH-PX (**i**), SOD (**j**), and CAT (**k**) concentrations in different groups (n = 5 for each group). **P < 0.01, ***P < 0.001, and ****P < 0.001, compared to the control

ROS generation (Fig. 3a), DNA damage (Fig. 3b), mitochondrial alterations (Fig. 3c), and cell membrane injury (Fig. 3d) were assessed in DFCs to investigate cellular oxidative stress levels. In the EVs + H_2_O_2_ group, hASC-EV treatment substantially lowered the expression of dichlorodihydrofluorescein (DCF; an ROS generation marker) (Fig. 3e) and 8-OHdG (a DNA damage marker) (Fig. 3f) and the concentration of malondialdehyde (MDA; a cell membrane injury marker) compared to those in the H_2_O_2_ group. The JC-1 fluorescent dye was employed to investigate mitochondrial alterations in H_2_O_2_-stimulated DFCs. The results revealed a considerable increase in the JC-1 ratio after hASC-EV treatment compared to that in the H_2_O_2_ group, indicating restoration of mitochondrial membrane potential (Fig. 3g). Next, the antioxidant activity of hASC-EVs was assessed using FRAP, glutathione peroxidase (GSH-PX), SOD, and CAT assays to further examine the suppressive influence of hASC-EVs on oxidative stress. The hASC-EV treatment increased the concentrations of FRAP (Fig. 3h), GSH-PX (Fig. 3i), SOD (Fig. 3j), and CAT (Fig. 3k) compared to those in the H_2_O_2_ group. Overall, these findings show that hASC-EV therapy reduces oxidative damage, increases antioxidant activity, and reduces oxidative reactivity in H_2_O_2_-stimulated DFCs.

### hASC-EVs alleviate H_2_O_2_-suppressed cell differentiation

**Fig. 4:**
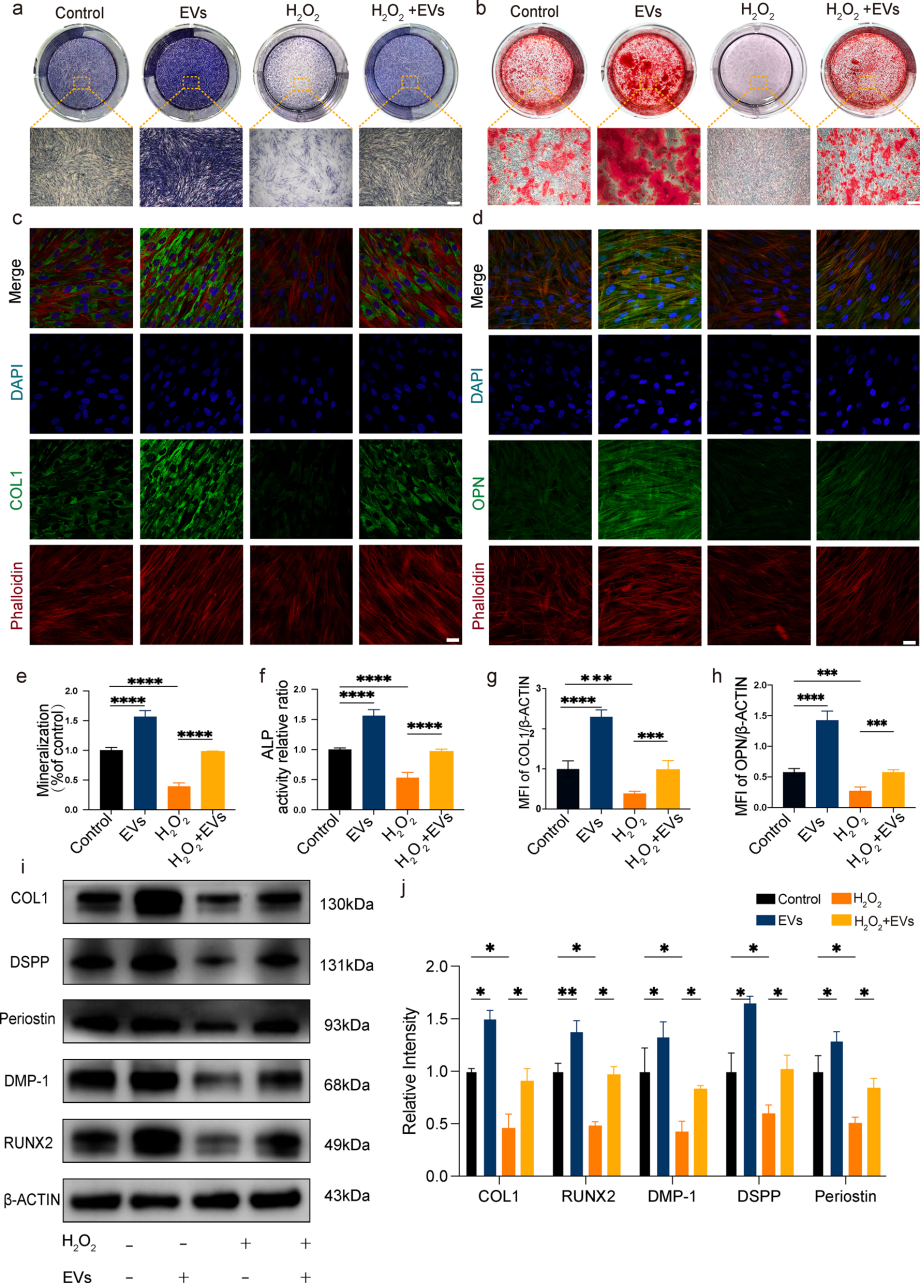
Human adipose-derived mesenchymal stem cell extracellular vesicles inhibit oxidative damage and promote dental follicle cell (DFC) osteogenesis and odontogenesis in an oxidative stress environment. Osteogenic differentiation was detected using **a** alkaline phosphatase (ALP) and **b** Alizarin red S staining (scale bar = 500 μm). **c**, **d** Immunofluorescence images displaying the manifestation of anti-COL1 and anti-OPN in DFCs (scale bar = 20 μm). Histograms showing quantification of the mineralization (**e**), ALP activity (**f**), mean fluorescence intensity (MFI) of COL1 (g), and MFI of OPN expression (**h**). **i** Western blot analysis of changes in the expression of odontogenesis- and osteogenesis-associated proteins. **j** Histogram illustrating the quantitative analysis of COL1, RUNX2, DMP-1, DSPP, and periostin protein expression in different groups. *P < 0.05, **P < 0.01, and ***P < 0.001, compared to the control

ALP (Fig. 4a) and ARS (Fig. 4b) staining revealed that hASC-EV treatment considerably boosted calcium deposition and ALP activity, but H_2_O_2_ treatment significantly reduced both processes as compared to those in the controls. Under the same H_2_O_2_-induced oxidative stress, the culture pretreated with hASC-EVs displayed higher levels of mineralized clusters and ALP activity than the untreated culture.

The immunofluorescence technique was utilized to observe alterations in the expression levels of different odontogenic indicators in the DFCs. In the hASC-EV groups, the expression levels of anti-OPN and COL1 were considerably elevated (Fig. 4c and d). Compared to the H_2_O_2_ group, hASC-EV treatment led to a considerable increase in the mean fluorescence intensity of OPN and COL1 (Fig. 4g and h). The expression of odontogenic biomarkers, including COL1, DSPP, periostin, DMP-1, and RUNX2, was examined to further investigate the effect of hASC-EVs on odontogenic development (Fig. 4i). In contrast to those of the control group, the protein levels of the aforementioned parameters increased in the hASC-EV groups but decreased markedly with the addition of H_2_O_2_. Moreover, the expression of these proteins was enhanced in the groups treated with both hASC-EVs and H_2_O_2_ compared to those treated with H_2_O_2_ alone (Fig. 4j). These findings indicate that hASC-EVs may preserve the osteogenic and odontogenic functions of hDFCs by protecting them from H_2_O_2_-induced damage, which is one of the most essential stemness properties of DFCs in vitro.

### Analysis of hASC-EVs and their specific miRNAs

**Fig. 5:**
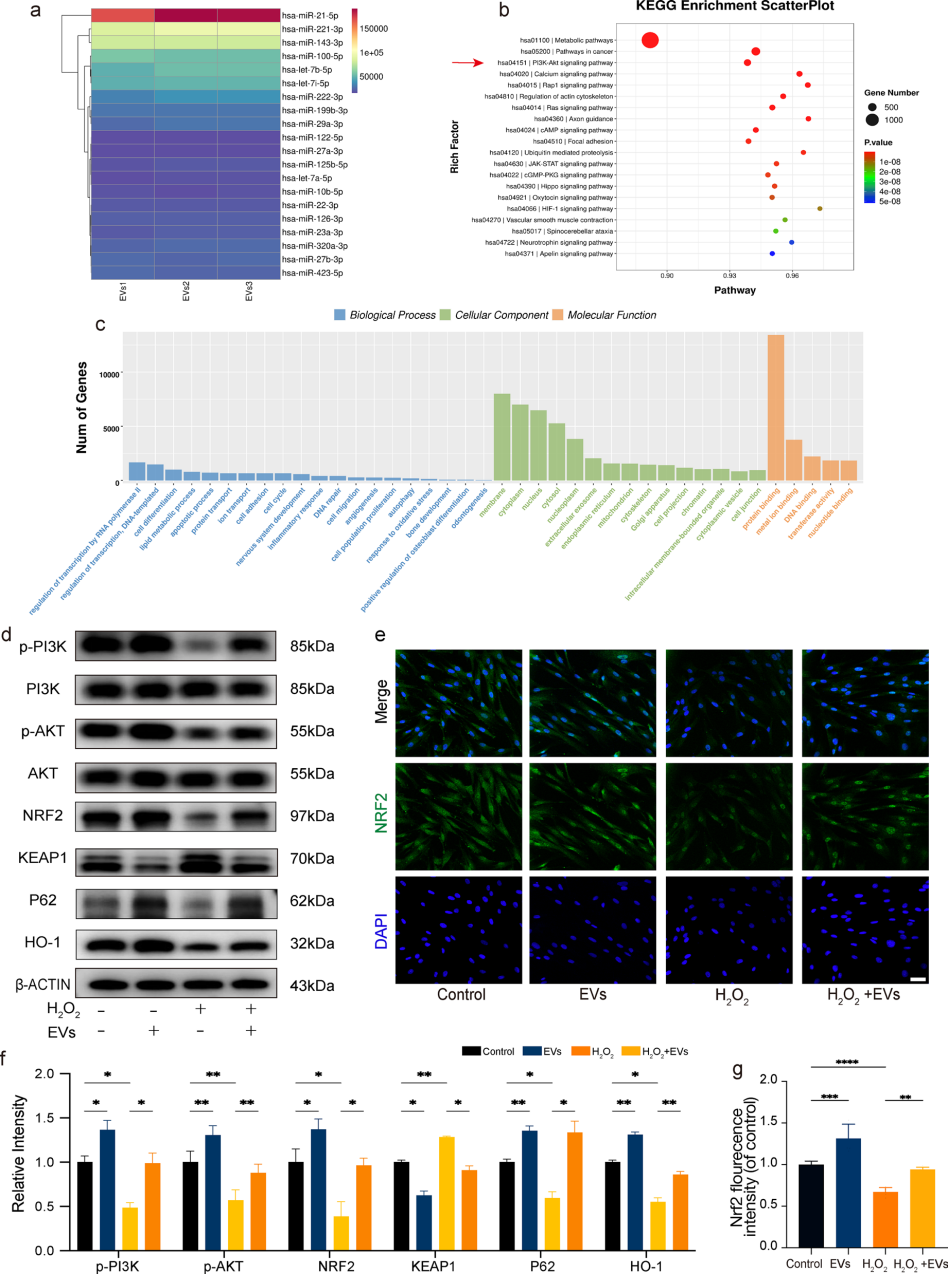
Human adipose-derived mesenchymal stem cell extracellular vesicles (hASC-EVs) regulate cellular protection from oxidative stress damage via PI3K/Akt/NRF2 signaling. **a** The top 20 miRNAs in hASC-EVs were examined using human miRNA sequencing. miRNA profiling and gene target analysis using the KEGG and GO databases were performed on the target genes of miRNAs enriched in hASC-EVs. **b** Significantly enriched KEGG pathways and **c** GO terms of biological processes (BPs), cellular components (CCs), and molecular functions (MFs). **d** Western blot results showing PI3K, p-PI3K, Akt, p-Akt, NRF2, KEAP-1, P62, and HO-1 protein expression in dental follicle cells (DFCs). **e** Representative images showing NRF2 expression in DFCs. Scale bar = 20 μm. **f** Histogram illustrating the quantitative analysis of p-PI3K, p-Akt, NRF2, KEAP1, P62, and HO-1 protein expression in different groups. **g** Quantification of NRF2 immunofluorescence intensity. *P < 0.05, **P < 0.01, and ***P < 0.001, compared to the control

A total of 1511 miRNAs were identified in hASC-EVs via RNA sequencing, and the top 20 expressed miRNAs are listed in Fig. 5a. Furthermore, we performed target gene prediction based on the obtained miRNAs, and the predicted target genes were subjected to KEGG (Fig. 5b) and GO (Fig. 5c) enrichment analyses. The top 20 pathways included metabolic, cancer, PI3K-Akt signaling, calcium signaling, and Rap1 signaling pathways. Numerous binding, transcription factor, and enzyme processes were among the indicated roles. miRNAs were expected to have the following cellular components: cytoplasm, nucleus, nucleoplasm, membrane, and external exosome. The expected biological processes included cell differentiation, cell migration, apoptosis process, autophagy bone development, inflammatory response, response to oxidative stress, and odontogenesis. The indicated molecular functions included protein binding, metal ion binding, DNA binding, and transferase activity. Figure 5a–c roughly illustrates the functions of the EV miRNAs and their associated verified target genes.

### hASC-EVs activate the PI3K/Akt/NRF2 pathway under oxidative stress

To enhance our comprehension of the mechanism by which hASC-EVs inhibit oxidation pathways, we employed western blotting to examine the participation of the PI3K/Akt/NRF2 pathway (Fig. 5d). Under identical conditions, the administration of hASC-EVs resulted in the augmentation of the expression levels of PI3K, Akt, and NRF2 proteins, both in their basal state and in their state of phosphorylation, which had been repressed by H_2_O_2_. Significantly, the protein expression of P62 and HO-1 was suppressed in the presence of oxidative stress. However, this repression was effectively counteracted following treatment with hASC-EVs. Furthermore, the application of hASC-EVs was found to induce the translocation of NRF2 in DFCs, as demonstrated by immunofluorescence experiments (Fig. 5e). Additionally, the mean fluorescence intensity of NRF2 significantly increased in the hASC-EV treatment group compared to that in the group treated with H_2_O_2_. These findings suggest that hASC-EVs can modulate the activity of NRF2 protein under oxidative stress.

**Fig. 6:**
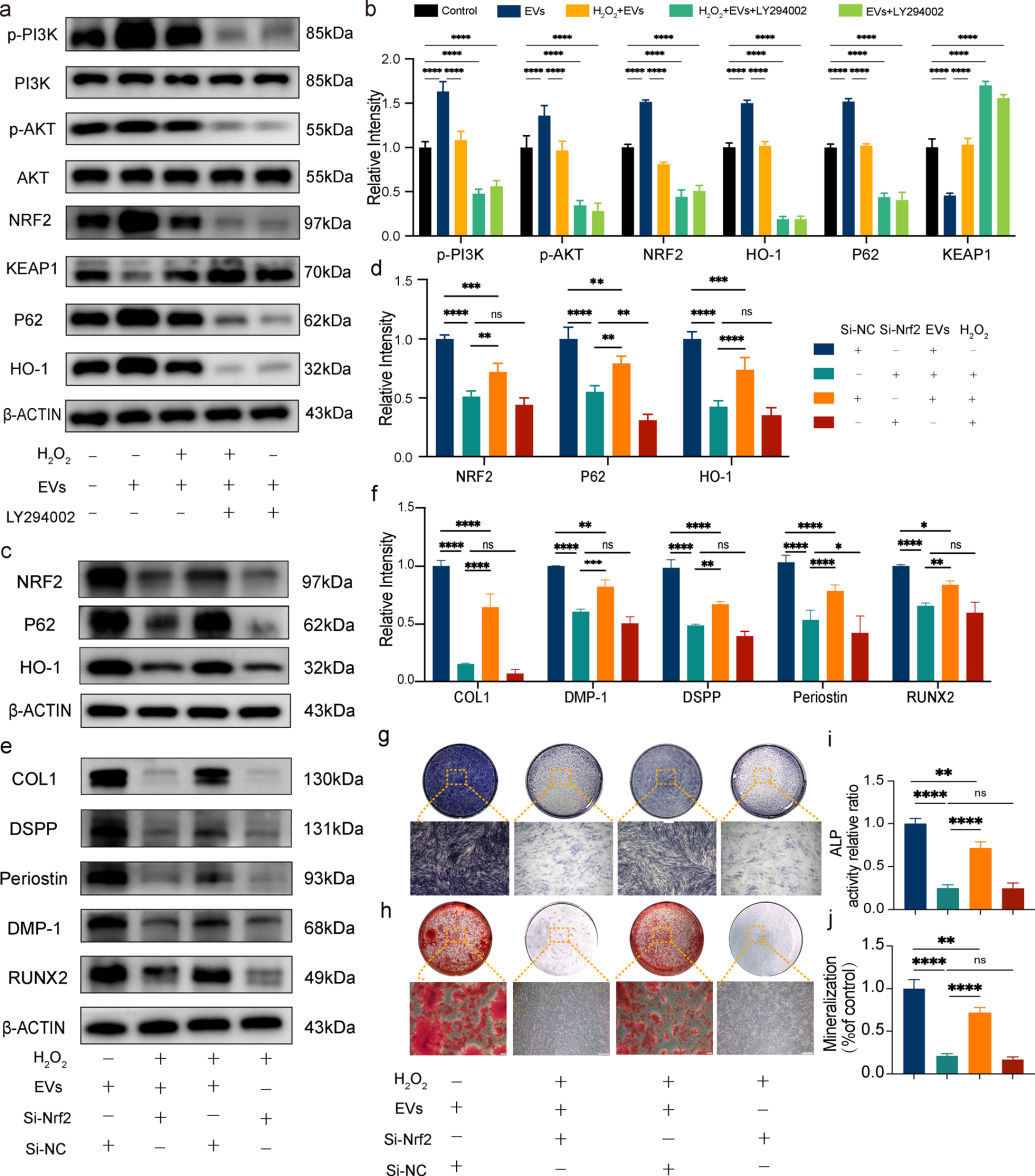
Human adipose-derived mesenchymal stem cell extracellular vesicles activate NRF2/HO-1 signaling via the PI3K/Akt pathway. **a** Western blot results showing PI3K, p-PI3K, Akt, p-Akt, NRF2, KEAP1, P62, and HO-1 protein expression in dental follicle cells pretreated with the PI3K/Akt inhibitor LY294002. **b** Histogram illustrating the quantitative analysis of p-PI3K, p-Akt, Keap-1, NRF2, and HO-1 protein expression after pretreatment with the PI3K/Akt inhibitor LY294002. **c** Changes in the NRF2 signaling pathway after *Nrf2* knockdown. **d** Histogram illustrating the quantitative analysis of NRF2, KEAP1, P62, and HO-1 protein expression in different groups. **e** Changes in the expression of odontogenesis- and osteogenesis-associated proteins after *Nrf2* knockdown. **f** Histograms illustrating the quantitative analysis of COL1, DSPP, periostin, DMP-1, and RUNX2 expression. **g** ALP and **h** ARS staining in different treatment groups after *Nrf2* knockdown. Scale bar = 500 μm. Histograms showing quantification of mineralization (**i**) and ALP activity (**j**) in different treatment groups after *Nrf2* knockdown. *P < 0.05, **P < 0.01, and ***P < 0.001, compared to the control

The administration of the PI3K/Akt inhibitor LY294002 completely suppressed the activation of PI3K and phosphorylation of Akt, leading to the abolishment of the protective effect exerted by hASC-EVs (Fig. 6a). Furthermore, the administration of LY294002 resulted in the suppression of NRF2, P62, KEAP1, and HO-1 expression induced by hASC-EV after 24 h of H2O2 treatment, as depicted in Fig. 6b. Subsequently, we conducted further experiments to examine the impact of LY294002 on the odontogenic and osteogenic differentiation of hASC-EVs under oxidative stress. The activity of ALP activity and mineralized nodule formation in DFCs treated with EVs + LY294002 were significantly decreased compared with those of the EVs group (Additional file 1: Fig. S4a-d). Additionally, the treatment of hASC-EVs did not counteract the inhibitory effects of H_2_O_2_ on the EVs + LY294002 + H_2_O_2_ group, when compared to the control group. Moreover, LY294002 significantly reversed the beneficial effects of hASC-EVs on the protein expression levels of COL1, DSPP, periostin, DMP-1, and RUNX2 in DFCs exposed to H_2_O_2_ (Additional file 1: Fig. S4e and f).Our results indicated that the activation of the NRF2/HO-1 pathway through the PI3K/Akt signaling pathway was the main mechanism by which hASC-EVs exerted their protective effects against oxidative stress.

### *Nrf2* knockdown reverses the protective effects of hASC-EVs against H_2_O_2_-induced oxidative stress

The investigation focused on elucidating the molecular mechanism by which hASC-EVs exert their effects on oxidative stress-induced damage, employing NRF2 siRNA. Western blot analysis results demonstrated that the administration of NRF2-targeting siRNA in DFCs led to a decrease in the expression levels of NRF2 and HO-1, as compared to those in the siNC group (Additional file 1: Fig. S2a). Following this, ARS (Additional file 1: Fig. S2b) and ALP (Additional file 1: Fig. S2c) staining techniques were employed to assess the impact of Nrf2 knockdown on the osteogenic differentiation of DFCs. The results revealed that Nrf2 knockdown significantly impeded the formation of mineralized nodules and ALP activity in DFCs, as compared to those in the siNC group.

The protective effect of hASC-EVs on dysfunction caused by oxidative stress in NRF2-siRNA DFCs was further investigated. Western blot analysis results demonstrated that there was not a substantial shift in the expression levels of NRF2, P62, and HO-1 in NRF2-siRNA DFCs treated with hASC-EVs following H_2_O_2_ stimulation (Fig. 6c and d). Additionally, the promotion of odontogenic and osteogenic differentiation of hASCs under oxidative stress was inhibited by *Nrf2* knockdown. Stimulation with H_2_O_2_ for 24 h led to a decrease in the odontogenic and osteogenic differentiation abilities of siNC-DFCs, while pretreatment with hASC-EVs before H_2_O_2_ treatment or co-treatment with hASC-EVs and H_2_O_2_ rescued their differentiation ability. In contrast, a decrease in the expression of COL1, DSPP, periostin, DMP-1, and RUNX2 (Fig. 6e and f), ALP activity (Fig. 6g and i), and calcium deposition (Fig. 6h and j) was observed in the siNRF2 hASC-EV group compared to those in the siNC hASC-EV group, indicating that the protective effects of both pretreatment and co-treatment with hASC-EVs on DFCs were diminished when *Nrf2* was knocked down.

### EV-hydrogel system characterization

**Fig. 7:**
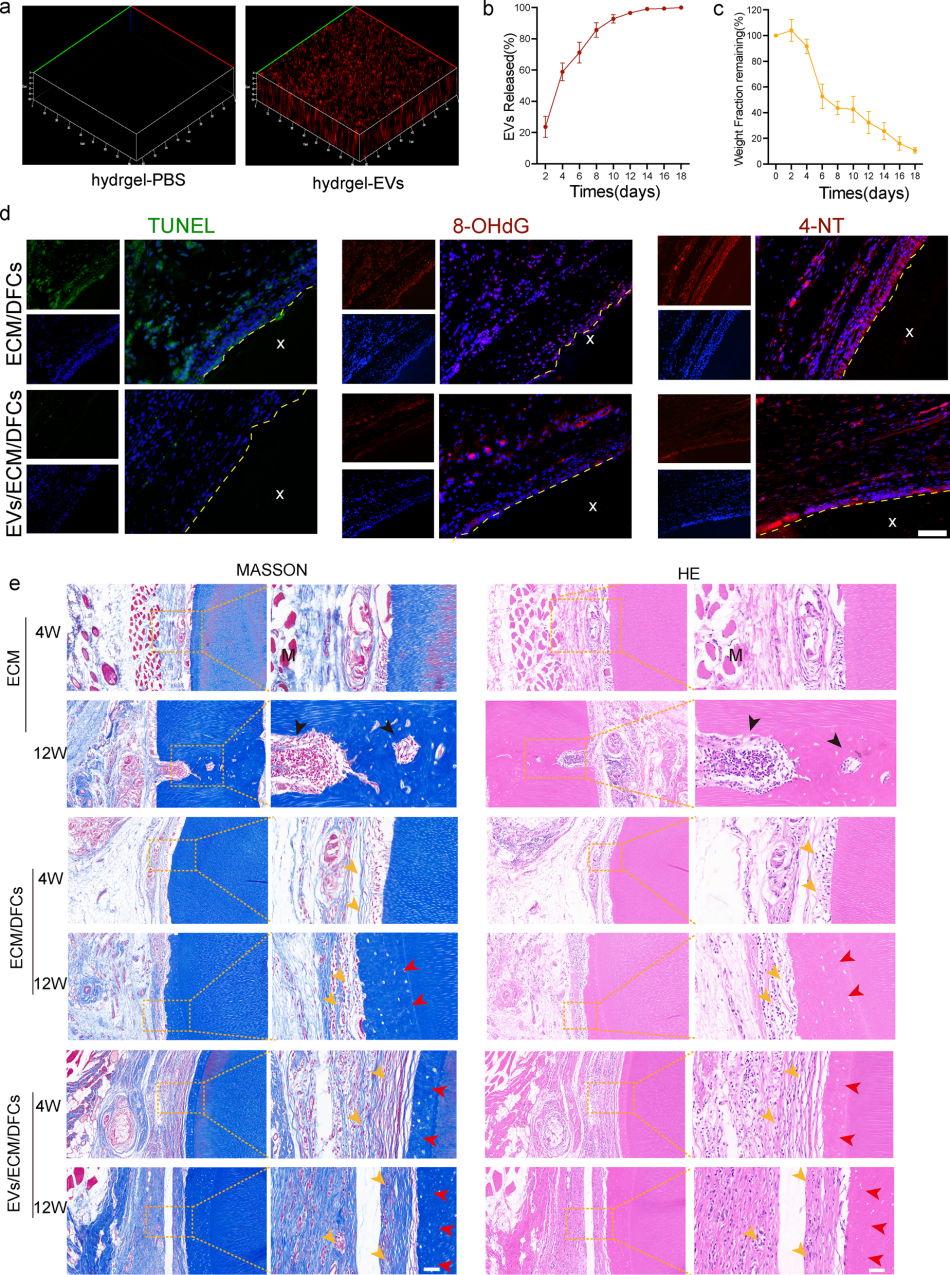
Hydrogel-loaded human adipose-derived mesenchymal stem cell extracellular vesicles envelope xenogeneic bio-root for subcutaneous grafting in Sprague–Dawley rats. **a** Three-dimensional (3D) distribution of extracellular vesicles (EVs; red fluorescence) in hydrogels. The line graph shows the 18-day release profile of EVs in hydrogels (n = 5 per group) (**b**) and weight loss degradation (**c**) in double-distilled water. **d** Immunofluorescence was used to assess cell apoptosis and oxidative damage after xenogeneic root transplantation. Scale bar = 50 μm. **e** Hematoxylin and eosin and Masson’s trichrome staining revealed tissue regeneration in Sprague–Dawley rats after subcutaneous abdominal transplantation of xenobiotic dental roots for 4 and 12 weeks. Yellow arrow: periodontal ligament-like fibers and new blood vessel tissue; red arrow: predentin; black arrow: absorption of xECM; M: muscle layer; X: xECM. Left images of each group = 100 μm; right images of each group = 50 μm

To ascertain the spatial arrangement of EVs within the hydrogel matrix, the EVs were subjected to labeling with PKH26 dye, which was subsequently incorporated into the hydrogel. PKH26-labeled EVs were evenly dispersed throughout the hydrogel (Fig. 7a). In vitro release profiles revealed a rapid initial delivery of EVs from the hydrogel in the first eight days, followed by a sustained release of > 95% for up to 18 days (Fig. 7b). Additionally, the residual weight ratio of the hydrogel stored in double-distilled water (DDW) at 37 °C was investigated. During the first four days, the weight of the EV hydrogel was effectively unchanged. A rapid degradation occurred during the 4^th^ and 6^th^ days, and the remaining weight slowly decreased to 10% over approximately 18 days (Fig. 7c).

### hASC-EVs improve the engraftment efficiency of ECM/DFC composites in vivo

To further confirm the mechanism underlying the hASC-EV-induced promotion of seed cells on the xECM surface, xenogeneic bio-roots treated with or without hASC-EVs were subjected to subcutaneous transplantation into the abdominal region of SD rats for 4 and 12 weeks (Additional file 1: Fig. S3d). Histochemical analysis of decalcified tissue sections revealed that in the first 4 weeks, hASC-EVs protected xenogeneic bio-roots against oxidative stress (Fig. 7d). Additionally, the TUNEL assay demonstrated that the apoptotic seed cells on the surface of the xECM exhibited green fluorescence.

The occurrence of apoptotic cells was significantly reduced in the bio-roots that were treated with hASC-EVs compared to that in the group treated with xECM/DFC. In addition, a gradual elevation in the levels of 3-NT and 8-OHdG was noted in xECM/DFCs. Conversely, hASC-EVs demonstrated a suppressive effect on the pathological changes occurring in the EVs/xECM/DFCs. These results indicate that hASC-EVs exhibit a comparable level of defense against oxidative-induced damage in living organisms.

Enthesis formation, encompassing the development of newly formed dentin and PDL structures, was observed on the surface of the xECM through the utilization of hematoxylin and eosin (H&E) and Masson’s trichrome staining techniques (Fig. 7e). In contrast to the xECM/DFC group, the EVs/xECM/DFC group exhibited a higher degree of density and alignment in the formation of PDL structures and neonatal dentin structures. In contrast, the xECM group did not demonstrate novel anatomical formations and was enveloped by the subcutaneous muscle layer and fascia. Twelve weeks after implantation, the xECM group showed obvious dissolution of the collagen matrix, accompanied by a large loss of dentin. Less collagen fiber encapsulation, newly regenerated angiogenesis revascularization, and neonatal dentin structure regeneration were detected in the xECM/DFC xenogeneic bio-roots implanted in SD rats. Conversely, upon treatment with EVs, the xenogeneic bio-root exhibited preserved structural integrity, with no observed dissolution or resorption of the collagen matrix. Numerous microvessels and PDL-tissues arranged perpendicular to the xECM were regenerated, which indicated that hASC-EVs could promote PDL-like and neonatal dentin structure regeneration in xenogeneic bio-roots.

**Fig. 8:**
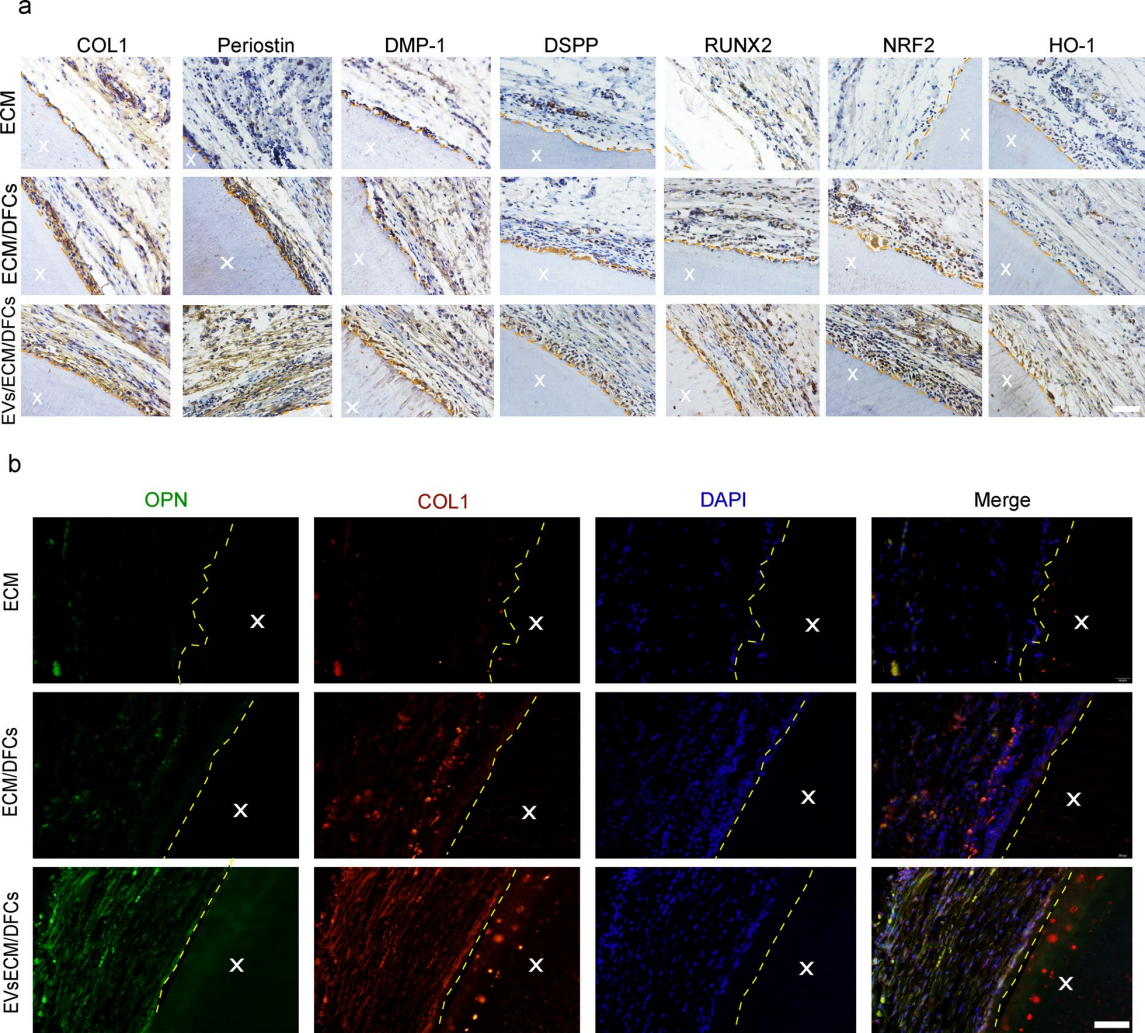
Human adipose-derived mesenchymal stem cell extracellular vesicles treatment inhibited oxidative stress and promoted periodontal ligament (PDL)-like tissue regeneration in xenogeneic bio-roots. **a** Immunohistochemical evaluation of odontogenic differentiation of bio-root composites in Sprague–Dawley (SD) rats. Scale bar = 50 μm. **b** Immunofluorescence was used to assess COL1 and OPN expression in bio-root composites. Scale bar = 50 μm. X: xenogeneic extracellular matrix

Immunofluorescence and immunohistochemistry were used to evaluate odontogenesis and osteogenesis in xenogeneic bio-root composites. Immunohistochemistry revealed that both the xECM/DFC and EVs/xECM/DFC groups expressed COL1 and periostin, the primary components of periodontal ligament fibers. The expression of dentin-specific proteins DSPP and DMP-1 was observed in the regenerated tissues of both experimental groups, indicating the successful formation of new dentin within the xECM. Mineralization in the developing predentin was indicated by the heightened expression of the osteogenic-related markers RUNX2 and COL1. The transplanted xenogeneic bio-roots without EV treatment exhibited significantly lower DSPP, DMP-1, COL1, periostin, and RUNX2 expression than that in the xenogeneic bio-roots subjected to EV treatment (Fig. 8a). In addition, immunofluorescence staining revealed relatively higher OPN and COL1 expression in the EVs/xECM/DFC group than in the other groups (Fig. 8b). Positively stained cells were distributed around the xenogeneic bio-roots. These results confirm that hASC-EVs can improve the heterogeneous environment in which bio-roots reside and promote the regeneration of heterogeneous bio-roots.

## Discussion

Dental implants are currently one of the primary methods for restoring tooth loss, and osseointegration is the criterion that determines implant success [42]. However, occlusal trauma and alveolar bone resorption can readily occur following implant restoration due to the absence of PDL tissue and periodontal proprioceptors [43]. Bio-root formation based on tissue-engineered stem cell/scaffold materials is projected to replace implants as a revolutionary treatment for tooth loss [4]. According to several studies, porcine tooth-derived dentin matrix, a type of xECM with a histological structure and bioactive factors comparable to those in human-derived dentin matrix, can promote odontogenesis and biomineralization [7]. xECM composite odontogenic stem cells have been widely used to construct bio-roots while regenerating dentin structure, dental pulp-like tissue, PDL, and proprioceptors for physiologically plausible functional healing [8, 9]. However, unfavorable oxidative stress is a substantial barrier to the success of xECM-based organ transplantation, raising the need for antioxidant capacity in xenograft regeneration [44].

xECM-based bio-roots exhibited aberrant oxidative stress and inflammation owing to immunological rejection, as seen in a previous study conducted with implanted xenografts [45]. Once the graft has been implanted, a significant number of inflammatory cells gather around it, resulting in a substantial accumulation of ROS and inflammation [14, 46]. These inflammatory factors and ROS increase the number of inflammatory cells at the graft site while also generating copious amounts of superoxide anion and H_2_O_2_ [15]. This cycle results in considerable ROS generation, which directly causes oxidative damage to DNA, proteins, and lipids and pathological changes to the tissue [47, 48]. To further detect the level of oxidative stress in cells, we can detect the expression of 8-OHdG, 3-NT, and MDA, which are biomarkers for DNA oxidative damage [49], protein oxidation [50], and lipid oxidative damage, respectively [51]. Here, the significantly increased expression of these biomarkers confirmed that xECM and H_2_O_2_ induced oxidative damage to seed cells.

In order to enhance the effectiveness of the implant, we intend to mitigate the oxidative stress-induced injury by the augmentation of antioxidant capacity in xenogeneic bio-root. In contrast to conventional antioxidants, MSC-EVs exhibit better biocompatibility and possess a greater capacity for efficient use inside the body, particularly in traversing the blood–brain barrier [52–54]. Consistent with a previous report, we found that hASC-EVs were absorbed by DFCs via the endocytic pathway and that they significantly improved cell function. MSC-EVs can protect cellular functions against oxidative stress-induced injury [24]. Xian et al. provided evidence that MSC-EVs have a protective effect on endothelial cells, shielding them from senescence induced by oxidative stress. Additionally, the researchers observed that MSC-EVs enhanced the migration of senescent cells and facilitated the restoration of tube formation [55, 56]. In the present study, we analyzed numerous functions to assess whether hASC-EVs promote the functional recovery of oxidative stress. The experimental results from proliferation, migration, and apoptosis assays provide evidence that the administration of hASC-EVs resulted in enhanced cellular activity, increased cell proliferation, facilitated cell migration, and decreased the occurrence of apoptotic cells in the presence of oxidative stress. Overall, EV treatment considerably improved and enhanced cellular function. Additionally, EVs can regulate the effects of cellular oxidative stress, including seizure-induced neuronal damage and UV-induced epidermis damage [56]. However, the efficacy of EVs against xenogeneic bio-root-induced oxidative stress is inadequately defined. In the present study, the significantly increased ROS generation and oxidative damage marker expression confirmed that xECM and H_2_O_2_ induced oxidative damage to seed cells. However, these effects were reversed by hASC-EV treatment. These results verified that EVs may regulate the oxidative stress levels of cells to a certain extent and inhibit oxidative stress-induced injury in xenogeneic bio-roots.

Mitochondria are one of the target organelles for oxidative damage [57]. As they are the center of cellular homeostasis, any damage to mitochondria disrupts intracellular metabolism and reduces the supply of ATP, which exacerbates cell damage and induces apoptosis [58, 59]. Several studies have shown that certain miRNAs in EVs, including miR-30 (ref. [60]) and miR-200a-3p [61], can act directly on mitochondria to regulate mitochondrial dysfunction [62]. Consistent with these findings, we found that hASC-EVs altered mitochondrial membrane potential via the intrinsic pathway. Through the evaluation of biomarkers and cellular antioxidant enzymes [63], we assessed the antioxidant capacity of DFCs. FRAP indicates the overall levels of antioxidants [64]. SODs are the primary enzymes that catalyze the conversion of superoxide anions to H_2_O_2_ (ref. [65]), while CAT and GSH-PXs further reduce H_2_O_2_ to water. GSH-PX conversion requires the coupled oxidation of GSH to glutathione disulfide [66]. Oxidative stress lowers the cellular concentrations of FRAP, GSH-PX, CAT, and SOD. Notably, our findings revealed that hASC-EV treatment elevated the levels of these markers compared to their levels in cells under oxidative stress, demonstrating the antioxidant properties of hASC-EVs in oxidative DFCs. These results verified that hASC-EVs can regulate the oxidative stress levels of cells to a certain extent.

In addition to cellular functions and oxidative stress levels, the differentiation capacity of DFCs plays a significant role in xenobiotic tooth root regeneration, which is directly affected by tissue regeneration. Multiple studies have demonstrated that MSC-EVs possess the ability to facilitate cellular differentiation and tissue regeneration to a limited degree, while concurrently modulating the oxidative stress milieu [67]. Consistent with previous research [68, 69], excessive ROS levels inhibited the ability of MSCs to differentiate multi-directionally. Under oxidative stress, we discovered that hASC-EV treatment improved the osteogenic differentiation of DFCs by promoting mineralized nodule formation, ALP activity, and COL1 and RUNX2 levels. ALP activity and COL1 levels can be used to evaluate early osteogenic differentiation [70, 71]. The formation of mineralized nodules, which can be identified using ARS staining, is indicative of late-phase osteogenic differentiation [70]. These results demonstrate that EVs can effectively enhance the osteogenic differentiation potential of cells, even in the presence of oxidative stress, bringing it close to the levels observed under normal conditions. Furthermore, we investigated the expression of odontogenic markers in addition to the modulation of osteogenic differentiation. This effect is achieved through the upregulation of key genes involved in odontogenesis, namely DSPP, DMP-1, and periostin. DSPP and DMP-1 serve as reliable indicators of newly synthesized dentin, while periostin and COL1 are distinct markers specific to PDL tissue [72]. Our in vivo and in vitro experiments provided additional evidence to support the notion that hASC-EVs influenced osteogenesis and played a role in the formation of dentin, specifically the predentin and the odontoblast layer, as well as the PDL tissues, particularly in the presence of oxidative stress conditions. In general, the experimental data we have obtained suggest that hASC-EVs have a significant impact on the regeneration of foreign root tissues and the differentiation of DFCs into odontogenic cells. This effect has been observed both in in vivo and in vitro.

NRF2/HO-1 signaling plays a crucial role in maintaining cellular redox homeostasis under conditions of oxidative stress [73]. The findings of our study indicated that hASC-EVs exerted a protective effect against oxidative stress-induced damage to the cellular functions of seed cells and tissue regeneration. This protective mechanism was mediated through the activation of the NRF2/ HO-1 pathway, which was involved in anti-oxidation processes [32, 74]. In the cellular model of oxidative stress induced by H_2_O_2_, prior exposure to hASC-EVs expedited the degradation of KEAP1, leading to the translocation of NRF2 and improving the expression of HO-1. This was subsequently accompanied by a decline in oxidative damage [75]. The activation of the NRF2 signaling pathway in DFCs had the potential to decrease the production of ROS, enhance the activity of antioxidant enzymes, and alleviate the negative effects of elevated ROS on osteogenic and odontogenic differentiation. Multiple signaling pathways, such as PI3K/Akt, AMPK, and MAPK, have been observed to induce the activation of NRF2 in different disease models. The cellular defense against oxidative injury can be facilitated through the interplay of the PI3K/Akt and NRF2/HO-1 signaling pathways [76, 77]. It has been verified that in line with the observed NRF2 activation pattern, the administration of hASC-EVs also lead to an elevation in the phosphorylation of PI3K/Akt. This suggested that both the NRF2/HO-1 pathway and the upstream PI3K/Akt pathway played a critical role in mediating the antioxidative impact of hASC-EVs.

NRF2, as a crucial transcription factor, can act against oxidative stress by activating numerous genes encoding cytoprotective and antioxidative enzymes [78–80]. Conversely, the inhibition of NRF2 through the use of siNRF2 may attenuate these protective effects [36, 81–83]. Subsequent investigations have demonstrated that the administration of hASC-EVs did not induce any significant changes in the protein expressions of NRF2 and HO-1, as well as the differentiation processes related to bone and tooth formation, when subjected to oxidative stress in si-NRF2 DFCs. These findings suggest that the therapeutic application of hASC-EVs is unable to alleviate the oxidative harm resulting from the suppression of NRF2 in DFCs. The findings of this study indicate that hASC-EVs have the potential to modulate oxidative stress in xenobiotic tooth roots through the activation of the NRF2/HO-1 signaling pathway.

In summary, we showed that hASC-EVs could serve as a potential nanotherapeutic agent for improving the microenvironment for xenogeneic bio-root grafting and a viable strategy for PDL-like tissue regeneration. In addition, hASC-EVs may protect the biological characteristics of DFCs by maintaining cell viability, promoting migration, reducing apoptosis, eliminating cellular ROS, reducing mitochondrial change and DNA damage, enhancing cellular capacities, and enhancing antioxidant capacities to protect against redox imbalances following exposure to unfavorable transplant microenvironments via the PI3K/Akt/NRF2 defense system.

## Materials and methods

### Isolation and identification of DFCs

The ethical approval for this study was obtained from the Medical Ethics Committee of the First Affiliated Hospital of Zhengzhou University (2022-KY-0016-002). Following the extraction of a human third molar, dental follicle cells (DFCs) were obtained from the remaining dental follicle tissue and cultured in alpha-modified Eagle's medium (α-MEM, Hyclone, Grand Island, USA) supplemented with 10% fetal bovine serum (FBS, Gibco, Grand Island, USA) and 1% penicillin streptomycin (P/S, Gibco). Passage 3 DFCs were distributed into a 12-well plate at a density of 5 × 10^4^/well and cultured with an adipogenic induction liquid and an osteogenic medium. The adipogenic induction liquid contained 10 nM dexamethasone, 0.5 mM isobutylmethylxanthine and 2 mM insulin (Sigma-Aldrich, St. Louis, MO, USA), while the osteogenic medium contained 10 mM β-glycerol phosphate, 50 mg/mL ascorbic acid, and 100 nM dexamethasone (Sigma). To evaluate specific cell surface molecules, the DFCs were subjected to incubation in the presence of anti-CD31, anti-CD34, anti-CD73, anti-CD90 and anti-CD105 antibodies (1:100, BD Biosciences, Franklin Lakes, NJ, USA). An Accrue C6 Flow cytometer (Becton, Dickinson and Company, New Jersey, USA) was used to identify cell groups.

### Isolation and characterization of hASC-EVs

The hASCs were obtained from Cyagen Biosciences Inc. (CA, USA) and cultured in Dulbecco’s modified Eagle medium (Hyclone) with 1% P/S and 10% FBS. Once the cell confluence reached 80%, the medium was replaced with exosome-free FBS (SBI, System Biosciences, San Francisco, CA, USA). Following a 48-h incubation period, the supernatant containing cellular secretions was collected in order to isolate EVs. The supernatants were subjected to rotary acceleration at 1000 ×*g* for a duration of 10 min, and subsequently at 3000 ×*g* for a duration of 20 min. This was followed by filtration through a 0.22 μm sterilised filter(Millipore, Darmstadt, Germany).Exosome Isolation™ (Invitrogen, Carlsbad, CA, USA) solution was used to separate the EVs from the supernatant. The EVs were characterized using transmission electron microscopy (TEM; Tecnai G2 Spirit 120 kV, USA), nanoparticle tracking analysis (Zeta View Particle Metrix PMX, Germany), and western blotting for CD63, TSG101, and HSP70. Single-particle interferometric images were quantified using the ExoView device (Nano View Biosciences, Boston, MA, USA). LC Sciences (USA) carried out miRNA sequencing on hASC-EVs, and the preparation of tagged miRNA sequencing libraries, sequencing, and subsequent next-generation sequencing data analysis were conducted.

### xECM fabrication

xECM was generated as previously described for human-treated dentin matrix [11], and small porcine incisors and lateral incisors were used as the source of xECM. Using a high-speed handpiece, we removed the covered enamel and periodontal tissue from the tooth surface. Then, we completely removed the pulp tissue and a tiny amount of pre-dentin from the inner wall of the pulp cavity. Finally, we continued processing the prepared dentin material and ground away the dentin evenly until it met the implantation requirements. The obtained porcine dentin matrices were shaped into cones with diameters of 3–5 mm and lengths of 7–10 mm (Additional file 1: Fig. S3a). The composites were mechanically cleaned in deionized water and treated with 17%, 10%, and 5% ethylene diamine tetra-acetic acid (EDTA, Sigma) for 20, 18, and 15 min, respectively. The xECM components were stored for 72 h at 4 °C in a-MEM. The utilization of scanning electron microscopy (SEM) was employed to examine the morphology and histology of the xECM (Additional file 1: Fig. S3b). The xECM was collected in accordance with the procedure proposed by the International Organization for Standardization (ISO10093). As previously mentioned, 20 g of pulverized xECM was combined with 100 mL a-MEM, and the mixture was kept at 37 °C for 3 d. The xECM extracts were collected and filtered through 0.22 μm for induction of compressive odontogenic and osteogenic differentiation in vitro.

### Intracellular uptake of EVs

EVs were identified with PKH26 fluorescent labeling (Sigma-Aldrich). The DFCs were subjected to incubation with PKH26-marked EVs for time frames of 3, 6, 12, 24, and 48 h at 37 °C. Following PBS washing, the nucleic acid component of the DFCs was labeled with DAPI (Sigma). The specimens were examined using a fluorescence microscope (Zeiss, Imager Z2, Oberkochen, Germany).

### Cell treatment

In order to elicit oxidative stress-induced damage in an in vitro setting, the DFCs were subjected to a treatment of 200 μM H_2_O_2_ for 24 h. In order to assess the impact of hASC-EVs on H_2_O_2_-induced DFCs, the samples underwent a 24 h pretreatment with hASC-EVs, followed by a 24 h stimulation with H_2_O_2_. After a 48-h incubation period, distinct cell groups were subjected to further experimental assays for analysis.

### Cell proliferation

Cell viability was determined using the Cell Counting Kit-8 (CCK-8) assay in 96-well plates containing 7000 cells per well. The DFCs were exposed to varying concentrations of H_2_O_2_ for 3, 6, and 12 h to determine the optimal H_2_O_2_ concentration for establishing a model of oxidative stress. Cell viability changes were observed using different concentrations of EVs co-cultured with cells for 24 h. To detect the protective effects of EVs on H_2_O_2_-induced DFCs, DFCs were pretreated with varying concentrations of EVs for 24 h and then exposed to 200 M H_2_O_2_ for 24 h to observe changes in cell activity. Then, 10% (v/v) CCK-8 medium was co-incubated with cells for 1 h to detect alterations in cell viability. Using a microplate scanner set at 450 nm, the optical density was calculated.

The ethynyldeoxyuridine (EdU, Ribo Bio, Guangzhou, China) assay was used to measure the percentage of DNA-replicating cells, which reflects the state of cell proliferation. With three independent samples for each treatment group, the ratio of the number of EdU-incorporated cells to the number of Hoechst-stained cells was used to determine the EdU incorporation rate. The specimens were visualised utilising an inverted fluorescence microscopy system (Olympus).

### Cell migration assays

The migration ability of DFC was assessed using a Transwell system (Corning, USA). Passage 3 DFCs were put in the top chambers of Transwell plates with low serum medium (0.5% FBS), and PBS, EV solution, or H_2_O_2_ were added to the lower compartment containing serum medium (10% FBS). After 24 h of co-cultivation, the cells in the top chamber were wrapped, and the cells in the bottom chamber were marked with 0.1% crystal violet (Sigma). The cells were enumerated in five random areas using an inverted microscopy system.

### Cell apoptosis analysis

The evaluation of cellular apoptosis was conducted by employing the TUNEL (Beyotime, China) and Annexin V-FITC/PI Apoptosis Detection kits (Beyotime). The TUNEL Apoptosis Detection kits were employed, as per the manufacturer's guidelines, to label the apoptotic cells present in H_2_O_2_-stimulated DFCs and xECM-induced tissue. TUNEL-positive cells showed red or green nuclear staining and observed using an inverted fluorescence microscope. Additionally, DFCs were collected and placed into a tube. Subsequently, the cells were treated with 5 µL of Annexin V-FITC and 10 µL of PI, and incubated for a duration of 20 min. Finally, the samples were analyzed using an Accrue C6 Flow cytometer.

### Intracellular ROS and mitochondrial membrane potential detection

ROS assay kit (Beyotime, China) and JC-1 staining were employed to measure intracellular ROS generation and mitochondrial membrane potential. The samples were subjected to incubation with a concentration of 10 M DCFH-DA in serum- and chemical-free cell culture solution for 30 min at 37 °C. JC-1 working solution was added with the sample and incubated for 20 min in the cellular incubator. The samples were observed with a fluorescence inverted microscope (Olympus, Japan).

### Detection of MDA and antioxidant enzyme activity

To detect intracellular antioxidant enzyme activity, the DFCs were collected into centrifuge tubes after different treatments with EVs and H_2_O_2_. The samples were lysed using ultrasound to collect supernatants for subsequent experiments. The samples and reaction reagents included those from the Lipid Peroxidation MDA Assay Kit (Dojindo) and the ferric ion reducing antioxidant power (FRAP), superoxide dismutase (SOD), and catalase (CAT) assay kits (Nanjing Jiancheng Bioengineering Institute, Nanjing, China). Each experiment was conducted on a microplate reader using the wavelength specified in the instructions.

### Immunofluorescence staining

Following a 15-min fixation in 4% paraformaldehyde, the samples were permeabilized with 0.1% Triton X-100 for 20 min. Each sample underwent treatment with a blocking agent and subsequent incubation with primary antibodies. The primary antibodies used were as follows: anti-CK14 (1:100; Cell Signaling Technology, Danvers, MA, USA), anti-c-Kit (1:400; Cell Signaling Technology), anti-STRO-1 (1:100; R&D Systems, Minneapolis, MN, USA), anti-Vimentin (1:500; Abcam, Cambridge, UK), anti-8-OHdG (1:100; Sigma), anti-collagen I (COL1; 1:100, Abcam), anti-osteopontin (OPN; 1:100, Abcam), and anti-NRF2 (1:100; Cell Signaling Technology). Following the retrieval of the antibodies, DAPI and the appropriate secondary antibody were incubated. Immunofluorescence images were acquired using a confocal microscope.

### Lentiviral transduction

*NRF2* stable knockdown (*NFE2L2*-RNAi) cells were established using Gene Chem (Shanghai, China) lentiviral particles. The RNAi sequence was 5′-CAGAGAAAGAATTGCCTGTAA-3′. As a negative control, lentiviral particles with scrambled sequences were used to carry out transduction (Sh-NC). The number of infections (MOI) was 40, and > 70% of the cells were successfully transferred. When the number of cells reached 80%, normal cells were used as controls, and 0.5 g/mL puromycin was used to pick out the transduced cells.

### Western blotting

To determine relative protein expression levels, EVs and cells were lysed using a protein extraction kit (Solarbio, Beijing, China). 20–30 µg of total protein from each group were added to each well; the proteins were subsequently undergoing electrophoretic separation through a 10% sodium dodecyl sulfate–polyacrylamide gel and then transferred onto polyvinylidene fluoride membranes. The membranes were treated with certain primary antibodies after being blocked with 5%BSA(Sigma) for 1 h. EV-related primary antibodies were detected with 1/500 diluted anti-HSP70, -CD63, and -TSG101. Other antibodies were detected with 1/1000 diluted NRF2, HO-1, KEAP-1, PI3K, p-PI3K, Akt, p-Akt, RUNX family transcription factor 2 (RUNX2), COL1, periostin, dentin sialophosphoprotein (DSPP), dentin matrix acidic phosphoprotein 1 (DMP-1),and β-Actin. After washing with TBST (50 mM Tris–HCl, pH 7.4, 100 mM NaCl, and 0.2% Tween-20), secondary antibodies (goat anti-mouse IgG and goat anti-rabbit IgG; 1:2000; Proteintech, China) were added and incubated with the membranes. The enhanced chemiluminescence system (Millipore) was utilized to observe the relative expression of the protein bands. The Western blotting procedure was replicated three times for each marker and analyzed using Image J software.

### ALP and ARS staining

The measurement of ALP activity was conducted using an ALP Kit (Solarbio). After the treatment, the cells were immobilized using a 4% paraformaldehyde solution and then stained using a working solution of ALP. In addition, the specimens were stained with 2% ARS (pH 4.2) to visualize calcification. Specimens were fixed in 4% paraformaldehyde, followed by staining with ARS for 15‒30 min. To quantify the mineral deposits, the samples were eluted with 10% cetylpyridinium and determined at the absorbance of 562 nm.

### Construction and characterization of the 3D-EV-hydrogel system

Vitro Gel (The Well Bioscience, USA) was diluted in a 1:3 v/v ratio with the Vitro Gel Dilution Solution. Dilution of EVs with PBS to a concentration of 0.5 μg/μl was initially performed. 50 μl of EVs was combined with 150 μl hydrogel for the experimental groups. An equivalent volume of hydrogel and PBS was employed as the control. To analyze the dispersion of EVs inside the hydrogel, PKH26 (Sigma-Aldrich) was used for labeling EVs, and laser confocal microscopy was employed to acquire images.

### Degradation and release of the 3D-EV-hydrogel system

Double-distilled water (DDW; 10 mL) was incorporated into the hydrogel system and incubated at 37 °C with agitation at 150 rpm. Samples of the release medium were taken every 2 d and stored in 1.5 mL conical containers before being promptly frozen at − 80 °C for further examination. A fresh aliquot of equivalent volume was introduced to maintain the overall volume. The concentrations of residual EVs were assessed using the bicinchoninic acid method. The EV-hydrogel system was maintained in aseptic glass receptacles containing 10 mL distilled deionized water. Subsequently, the vials were allowed to rotate at 150 rpm and 37 °C. DDW was aspirated every 2 d, and the weight of the EV hydrogel was calculated as Wt. The residual weight was calculated as a percentage, given by Wt/W0 × 100%, where W0 is the initial weight of the 3D-EV-hydrogel system.

### Subcutaneous implantation of xenogeneic bio-roots in SD rats

As previously described, xECM was structured into a scaffold about a 4 mm internal diameter and a height ranging from 5 to 7 mm (Additional file 1: Fig. S3a). rDFCs were seeded onto the xECM in 6-well plates at an amount of 1 × 10^5^ cells per well, and they were cultivated for 7 d after the initial planting. The xECM and rDFCs were then combined to create the xenogeneic bio-root complex (xECM/rDFCs). The xECM and xECM/rDFCs were used for implantation in vivo. For this experiment, the 3D-EV-hydrogel system was injected into the xECM and xenogeneic bio-root complex, followed by a rapid incubation at 37 °C for a duration of 30 min (Fig. 5b). The SD rats were assigned to three groups (xECM, xECM/DFCs, and xECM/DFCs/EVs), each containing four to five rats. Following induction of anesthesia with 2% pentobarbital sodium (3 mL·kg^−1^) and Zoolitic (50 mg·kg^−1^), xenogeneic bio-roots with or without EVs were transplanted subcutaneously along their abdominal midlines. After either 4 or 12 weeks, the rats were euthanized, and their grafts and surrounding tissues were removed. The bio-roots were immersed in a 4% paraformaldehyde solution for subsequent analysis. Following this, they underwent decalcification using a 10% buffered EDTA solution (pH 7.4) for a period exceeding three months before being embedded in paraffin. Paraffin sections were prepared in conjunction with the longitudinal axis of the bio-root for subsequent histological analysis. This analysis involved staining the sections with hematoxylin and eosin (H&E), Masson’s trichrome, as well as utilizing immunofluorescence and immunohistochemical stains. The antibodies used for immunofluorescence detection included 8-OHdG, 3-nitrotyrosine (3-NT), COL1, and OPN. For the immunohistochemical assay, antibodies against DMP-1, DSPP, COL-1, OCN, RUNX2, periostin, NRF2, and HO1 were used.

### Statistical analysis

The data are presented in terms of the mean and standard deviation of the values obtained from separate experiments. The statistical analysis employed a two-tailed unpaired Student's t-test to compare two groups, while a one-way analysis of variance (ANOVA) was utilized to compare multiple groups. The observed outcomes were deemed to be statistically significant at various levels of significance, namely *P < 0.05, **P < 0.01, ***P < 0.001, and ****P < 0.0001. The statistical analyses were performed using GraphPad Prism V 9.0 (GraphPad Software, San Diego, CA, USA).

### Supplementary Information

**Additional file 1:** **Figure S1.** Construction of xenogeneic bio-root. **a** The fabricated xECM with an internal diameter of 4 mm and a height of 5–7 mm. **b** SEM appearance of xECM demonstrating exposed dentinal tubules. **c** Microscopic appearance show that xenogeneic bio-root complex was constructed in vitro with abundantly rDFCs covering over xECM surface at high density on day 7. **d** micro-CT showed that the xenogeneic root was located subcutaneously in the abdomen of the rat. **Figure S2.** Characterization of DFCs. **a** After dental follicle tissue culture for 3 days, polygonal cells spread out in the culture flask. P3 generation cells were polygonal—shaped. Mineralized nodules and lipid droplets were found when DFCs were cultured in osteogenic or adipogenic medium respectively. **b** DFCs were positive for Vimentin, STRO-1 and C-Kit and negative for CK14. **c** Flow cytometric analyses showed that DFCs were positive for CD73 and CD90 and negative for CD31 and CD34. The experiments were performed in triplicates. **Figure S3.** *Nrf2* knockdown on DFCs. **a** Western blot showing the protein levels of NRF2 and HO-1 in DFCs transfected with siNRF2 and siNC. **b** ARS and **c** ALP after *Nrf2* knockdown. Scale bar = 500 μm. The histograms represent the expression of NRF2 (**d**) and HO-1 (**e**). Histograms showing the quantification of mineralization (f) and ALP activity (**g**) after *Nrf2* knockdown. **Figure S4.** The effect of LY294002 on odontogenic and osteogenic differentiation of hASC-EVs under oxidative stress. **a** ARS and **b** ALP staining in different treatment groups pretreated with the PI3K/Akt inhibitor LY294002. Scale bar = 500 mm. Histograms showing quantification of mineralization (**c**) and ALP activity (**d**) in different treatment groups. **e** Western blot results showing COL1, DSPP, periostin, DMP-1, and RUNX2 protein expression in DFCs pretreated with the PI3K/Akt inhibitor LY294002. **f** Histograms illustrating the quantitative analysis of COL1, DSPP, periostin, DMP-1, and RUNX2 expression. *P < 0.05, **P < 0.01, and ***P < 0.001, compared to the control. **Figure S5.** The original Western blot data for hASCs and hASC-EVs. **1** Calnexin (100kda), **2** HSP70 (97kda), **3** TSG101 (45kda), **4** CD63 (70-35kda), **5** β-actin (43kda).

## Data Availability

All data are included in this published article and its Additional information files.
