# Correction: Mesenchymal stem cells‑derived extracellular vesicles protect against oxidative stress‑induced xenogeneic biological root injury via adaptive regulation of the PI3K/Akt/NRF2 pathway

**DOI:** 10.1186/s12951-023-02291-6

**Published:** 2024-01-22

**Authors:** Haojie Fu, Lin Sen, Fangqi Zhang, Sirui Liu, Meiyue Wang, Hongyan Mi, Mengzhe Liu, Bingyan Li, Shumin Peng, Zelong Hu, Jingjing Sun, Rui Li

**Affiliations:** 1 Department of Stomatology, The First Afliated Hospital of Zhengzhou University, Zhengzhou, 45000 China; 2 Academy of Medical Sciences at Zhengzhou University, Zhengzhou, 45000 China

**Correction to: Journal of Nanobiotechnology (2023) 21:466**

10.1186/s12951-023-02214-5

Following the publication of the original article, the authors reported that an additional file was not updated during the production process. Now, the author has provided Fig S4 in the supplementary material.

The original article [1] has been corrected.

### Electronic supplementary material

Below is the link to the electronic supplementary material.

Supplementary Material 1
