# Supplementary material for: Mesenchymal stem cells-derived extracellular vesicles protect against oxidative stress-induced xenogeneic biological root injury via adaptive regulation of the PI3K/Akt/NRF2 pathway
Source: J Nanobiotechnology. 2023 Dec 4;21:466. doi: 10.1186/s12951-023-02214-5 (PMC10696851; doi:10.1186/s12951-023-02214-5)

Additional file

**Fig. S1.** **Construction of xenogeneic bio-root.**


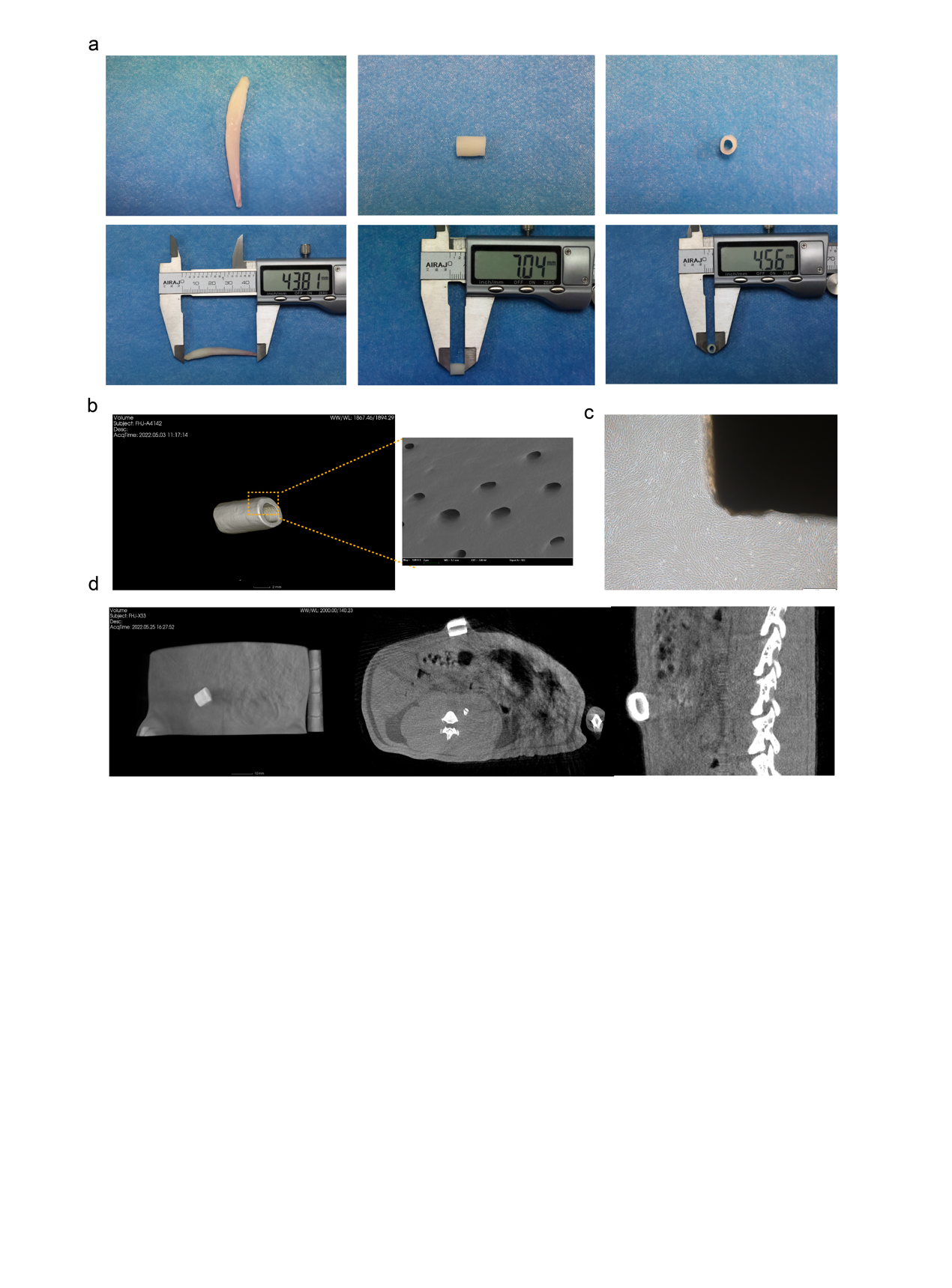


(a)The fabricated xECM with an internal diameter of 4 mm and a height of 5–7 mm. (b) SEM appearance of xECM demonstrating exposed dentinal tubules. (c) Microscopic appearance show that xenogeneic bio-root complex was constructed in vitro with abundantly rDFCs covering over xECM surface at high density on day 7. (d) micro-CT showed that the xenogeneic root was located subcutaneously in the abdomen of the rat.

**Fig. S2.** **Characterization of DFCs.**
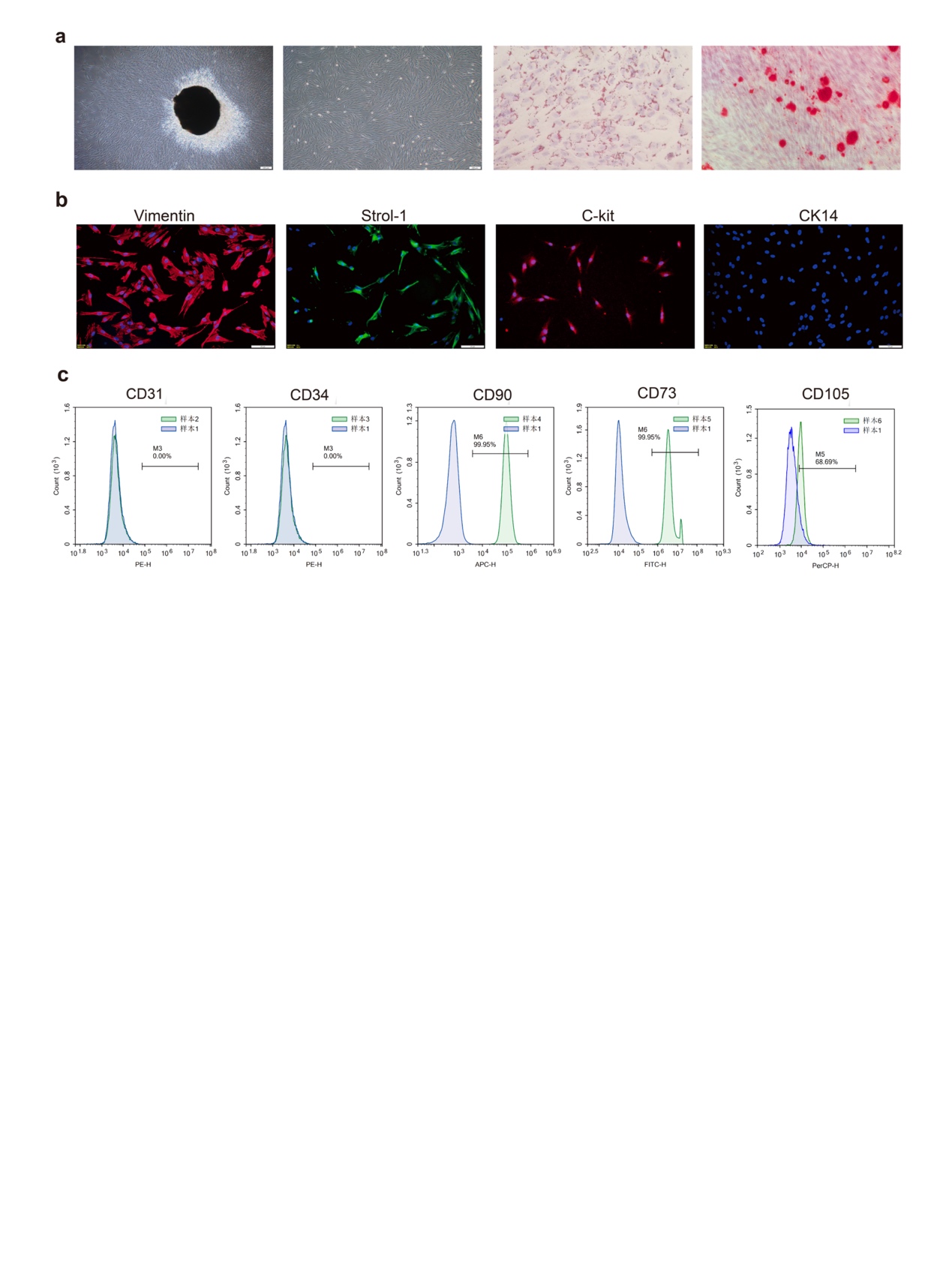


(a)After dental follicle tissue culture for 3 days, polygonal cells spread out in the culture flask. P3 generation cells were polygonal - shaped. Mineralized nodules and lipid droplets were found when DFCs were cultured in osteogenic or adipogenic medium respectively. (b) DFCs were positive for Vimentin、STRO-1 and C-Kit and negative for CK14. (c) Flow cytometric analyses showed that DFCs were positive for CD73 and CD90 and negative for CD31 and CD34. The experiments were performed in triplicates.

**Fig. S3. *Nrf2* knockdown on DFCs.**


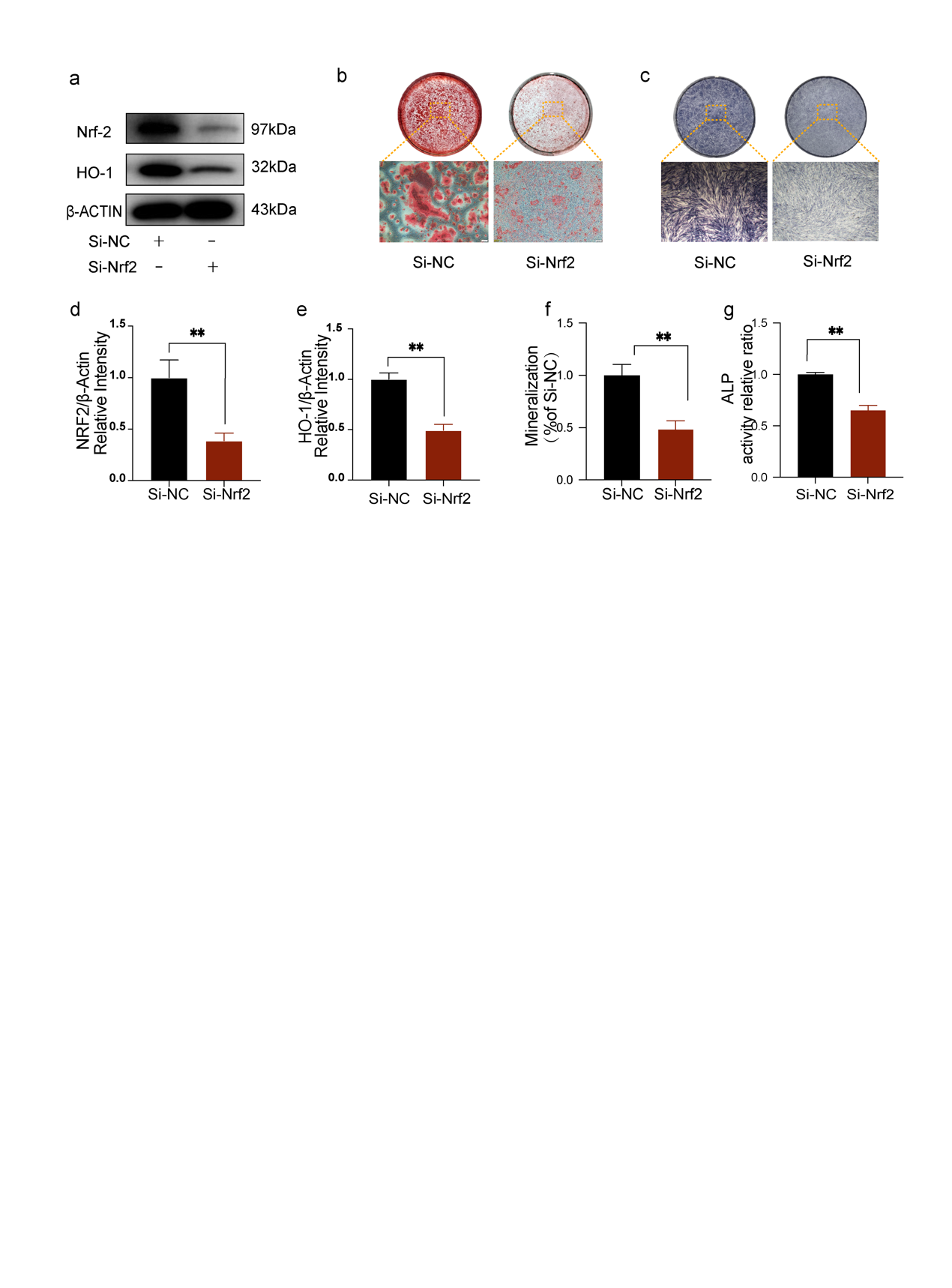


(a) Western blot showing the protein levels of NRF2 and HO-1 in DFCs transfected with siNRF2 and siNC. (b) ARS and (c) and ALP after *Nrf2* knockdown. Scale bar =500 μm. The histograms represent the expression of NRF2(d) and HO-1(e). Histograms showing the quantification of mineralization (f) and ALP activity (g) after *Nrf2* knockdown.

**Figure S4. The effect of LY294002 on odontogenic and osteogenic differentiation of hASC-EVs under oxidative stress.**


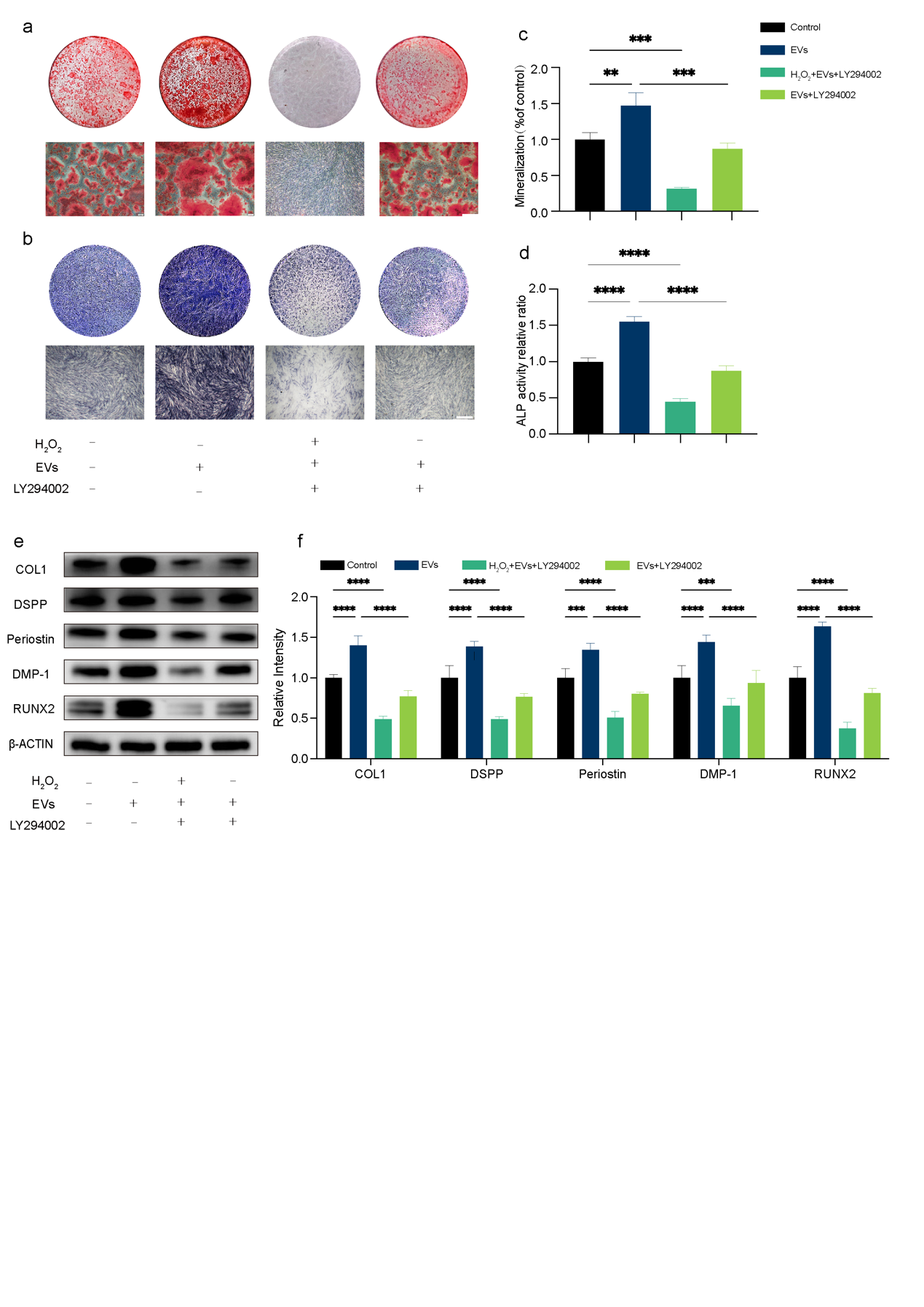


(a) ARS and (b) ALP staining in different treatment groups pretreated with the PI3K/Akt inhibitor LY294002. Scale bar = 500 mm. Histograms showing quantification of mineralization (c) and ALP activity (d) in different treatment groups (e) Western blot results showing COL1, DSPP, periostin, DMP-1, and RUNX2 protein expression in DFCs pretreated with the PI3K/Akt inhibitor LY294002. (f) Histograms illustrating the quantitative analysis of COL1, DSPP, periostin, DMP-1, and RUNX2 expression. * indicates P < 0.05, ** indicates P < 0.01, and *** indicates P < 0.001, compared to the control.

**Figure S5. The original Western blot data for hASCs and hASC-EVs.**

1. Calnexin (100kda)


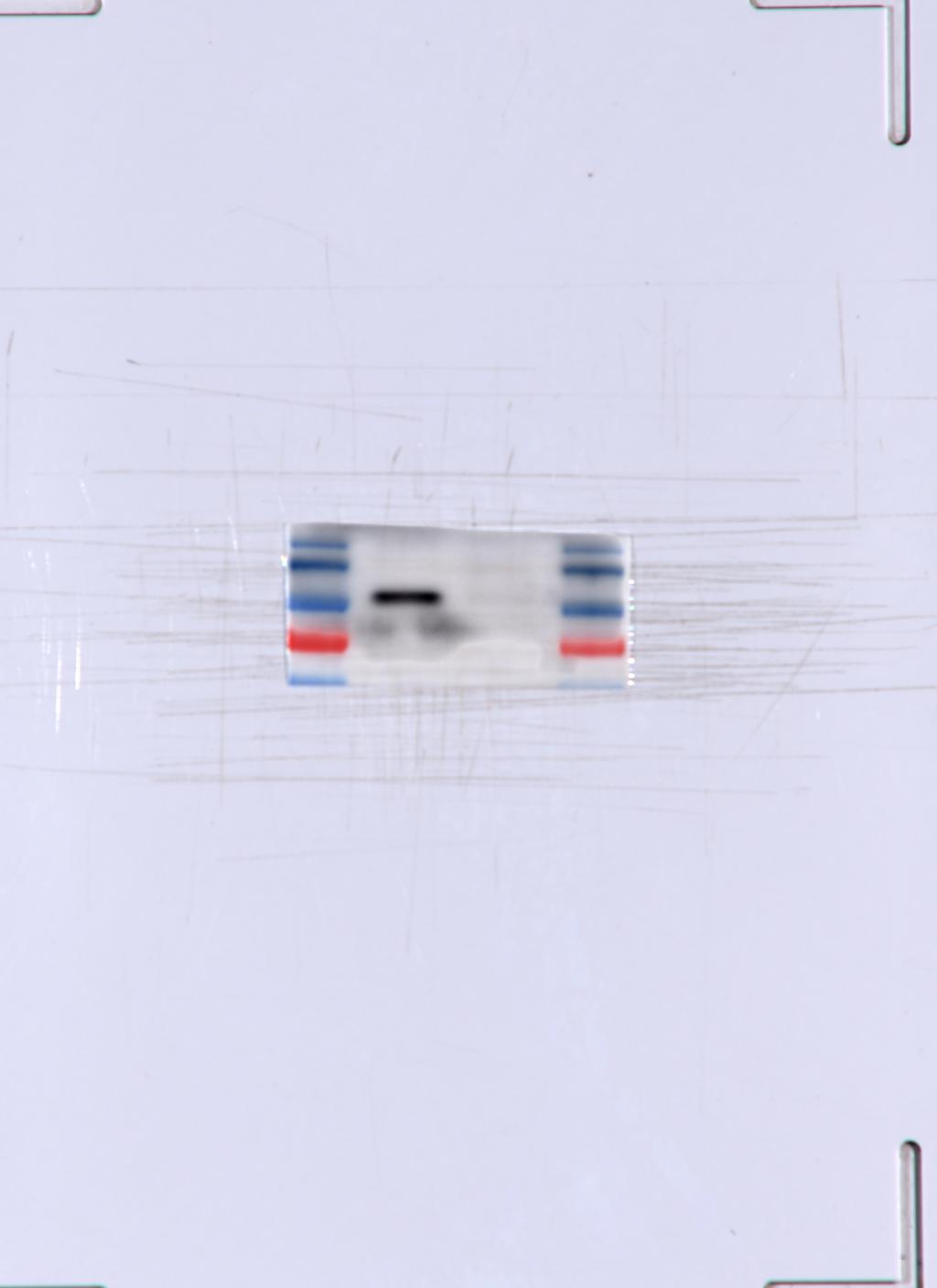


1. HSP70 (97kda)


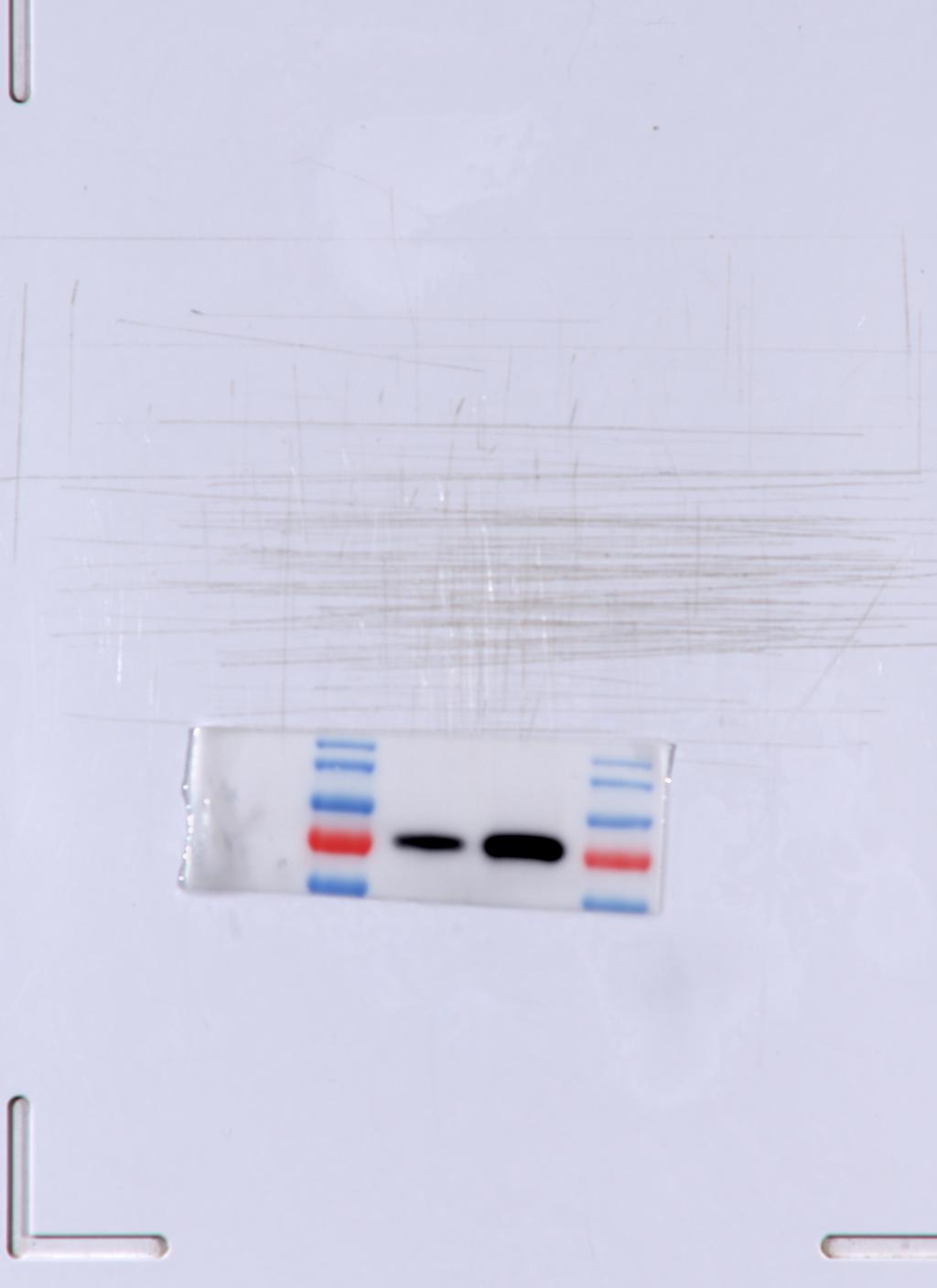


1. TSG101 (45kda)


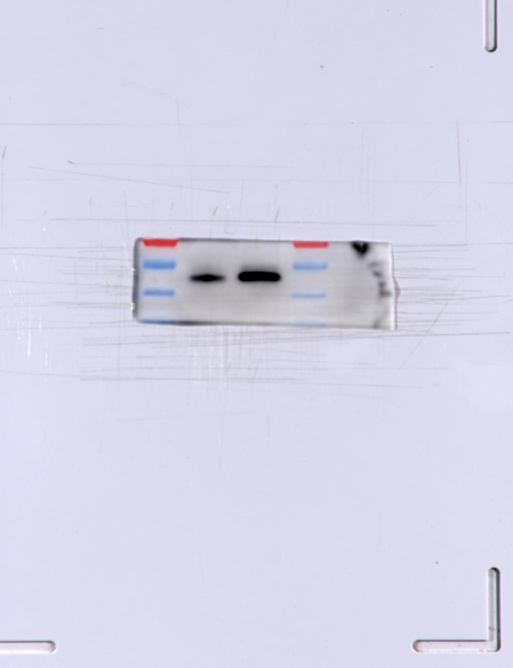

4. CD63 (70-35kda)


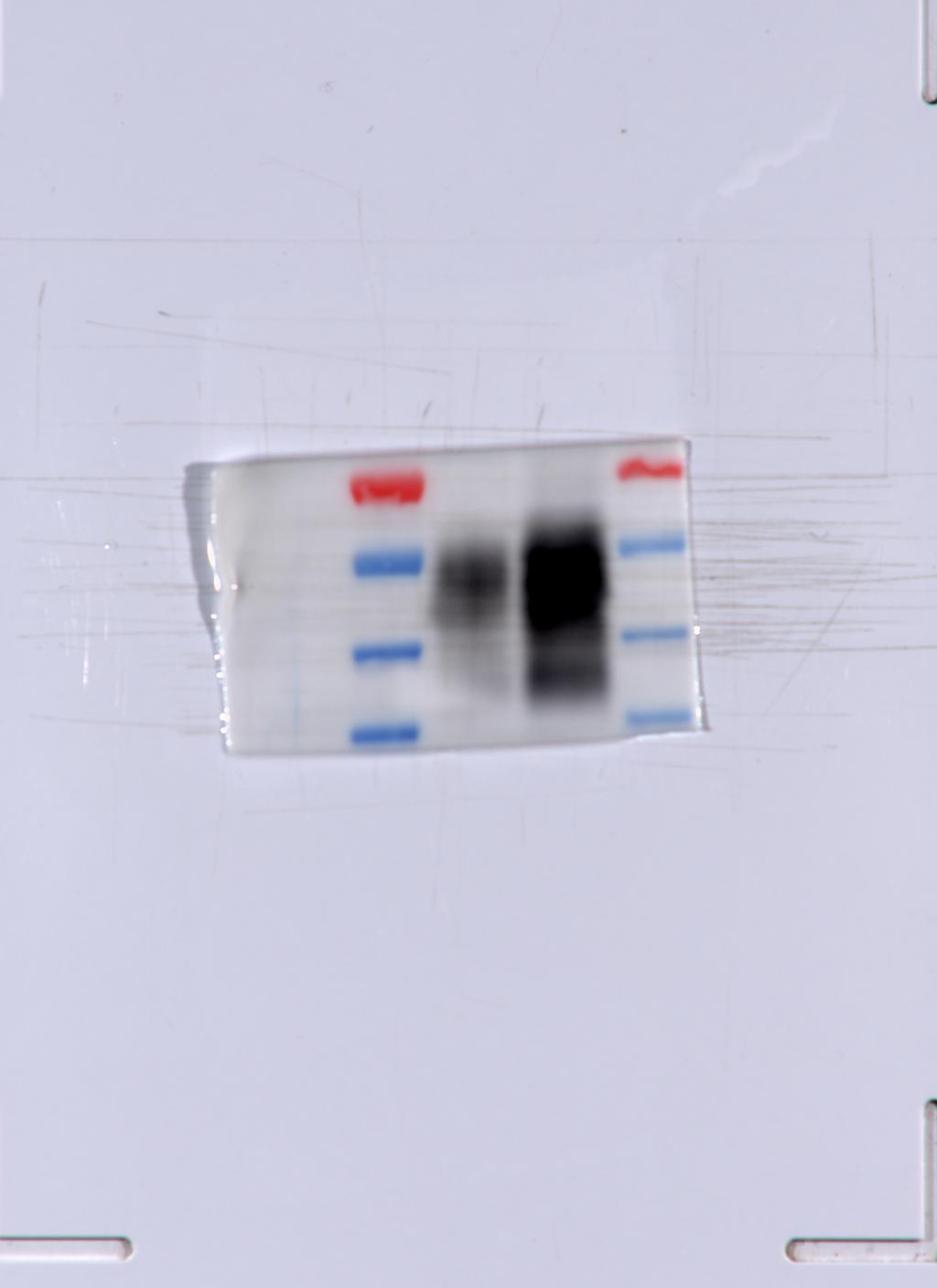


5. β-actin (43kda)


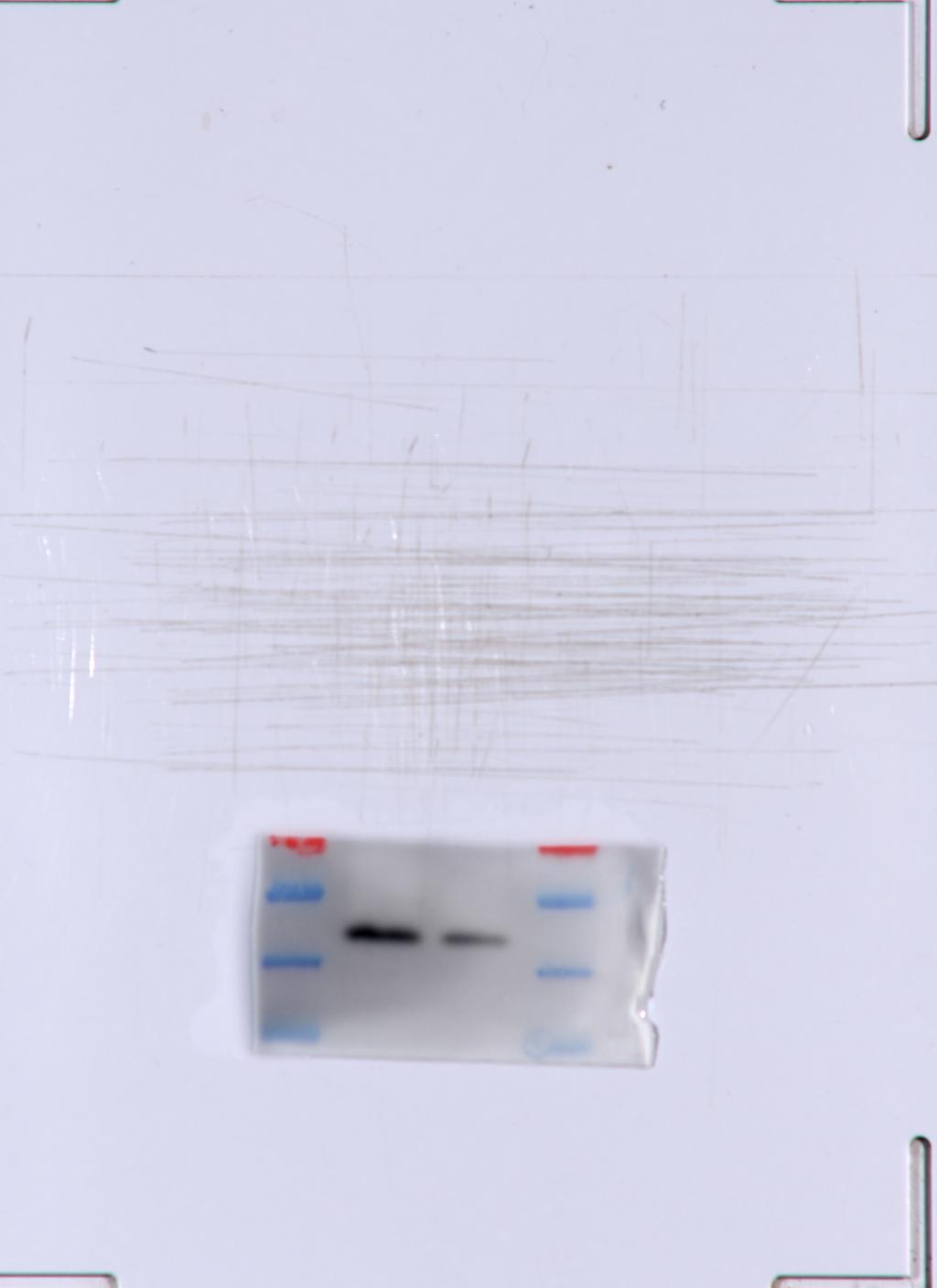

Supplement: Supplementary file 1 — Additional file 1: Figure S1. Construction of xenogeneic bio-root. a The fabricated xECM with an internal diameter of 4 mm and a height of 5–7 mm. b SEM appearance of xECM demonstrating exposed dentinal tubules. c Microscopic appearance show that xenogeneic bio-root complex was constructed in vitro with abundantly rDFCs covering over xECM surface at high density on day 7. d micro-CT showed that the xenogeneic root was located subcutaneously in the abdomen of the rat. Figure S2. Characterization of DFCs. a After dental follicle tissue culture for 3 days, polygonal cells spread out in the culture flask. P3 generation cells were polygonal—shaped. Mineralized nodules and lipid droplets were found when DFCs were cultured in osteogenic or adipogenic medium respectively. b DFCs were positive for Vimentin, STRO-1 and C-Kit and negative for CK14. c Flow cytometric analyses showed that DFCs were positive for CD73 and CD90 and negative for CD31 and CD34. The experiments were performed in triplicates. Figure S3. Nrf2 knockdown on DFCs. a Western blot showing the protein levels of NRF2 and HO-1 in DFCs transfected with siNRF2 and siNC. b ARS and c ALP after Nrf2 knockdown. Scale bar = 500 μm. The histograms represent the expression of NRF2 (d) and HO-1 (e). Histograms showing the quantification of mineralization (f) and ALP activity (g) after Nrf2 knockdown. Figure S4. The effect of LY294002 on odontogenic and osteogenic differentiation of hASC-EVs under oxidative stress. a ARS and b ALP staining in different treatment groups pretreated with the PI3K/Akt inhibitor LY294002. Scale bar = 500 mm. Histograms showing quantification of mineralization (c) and ALP activity (d) in different treatment groups. e Western blot results showing COL1, DSPP, periostin, DMP-1, and RUNX2 protein expression in DFCs pretreated with the PI3K/Akt inhibitor LY294002. f Histograms illustrating the quantitative analysis of COL1, DSPP, periostin, DMP-1, and RUNX2 expression. *P < 0.05, **P < 0.01, and *** [file 12951_2023_2214_MOESM1_ESM.docx]
